# Structural insight into the ligand binding mechanism of aryl hydrocarbon receptor

**DOI:** 10.1038/s41467-022-33858-w

**Published:** 2022-10-20

**Authors:** Shuyan Dai, Lingzhi Qu, Jun Li, Ye Zhang, Longying Jiang, Hudie Wei, Ming Guo, Xiaojuan Chen, Yongheng Chen

**Affiliations:** 1 Department of Oncology, NHC Key Laboratory of Cancer Proteomics & State Local Joint Engineering Laboratory for Anticancer Drugs, National Clinical Research Center for Geriatric Disorders, Xiangya Hospital, Central South University, Changsha, Hunan 410008 China; 2 Institute of Clinical Medicine, The First Affiliated Hospital of University of South China, Hengyang, Hunan 421001 China

**Keywords:** X-ray crystallography, Cell signalling, Enzyme mechanisms

## Abstract

The aryl hydrocarbon receptor (AHR), a member of the basic helix–loop–helix (bHLH) Per–Arnt–Sim (PAS) family of transcription factors, plays important roles in regulating xenobiotic metabolism, cellular differentiation, stem cell maintenance, as well as immunity. More recently, AHR has gained significant interest as a drug target for the development of novel cancer immunotherapy drugs. Detailed understanding of AHR-ligand binding has been hampered for decades by the lack of a three-dimensional structure of the AHR PAS-B domain. Here, we present multiple crystal structures of the *Drosophila* AHR PAS-B domain, including its apo, ligand-bound, and AHR nuclear translocator (ARNT) PAS-B-bound forms. Together with biochemical and cellular assays, our data reveal structural features of the AHR PAS-B domain, provide insights into the mechanism of AHR ligand binding, and provide the structural basis for the future development of AHR-targeted therapeutics.

## Abstract (web summary)

The aryl hydrocarbon receptor (AHR) plays important roles in regulating xenobiotic metabolism, cellular differentiation, and immunity. Here, the authors present multiple crystal structures of the *Drosophila* AHR PAS-B domain, providing insights into the mechanism of AHR ligand binding

## Introduction

After more than fifty years of intensive research, the aryl hydrocarbon receptor (AHR) has been established as a ligand-activated transcription factor that plays a critical role in various physiological processes, including xenobiotic metabolism, cell proliferation and development^1,2^. Recently, it has gained renewed attention for its key regulatory roles in the immune system and cancer immunology. Accumulating evidence has shown that AHR expression is upregulated in most tumor cells and is closely associated with tumor proliferation, invasion, metastasis, and immune escape^3–5^. In the tumor microenvironment, the activation of AHR induces immune tolerance of dendritic cells and promotes the differentiation and proliferation of regulatory T cells, leading to tumor immune escape and malignant proliferation^6–8^. Although a few studies have demonstrated its antitumorigenic roles^9^, the combination of AHR antagonists and immune checkpoint inhibitors is considered to have great potential in tumor immunotherapy^10,11^.

**Fig. 1:**
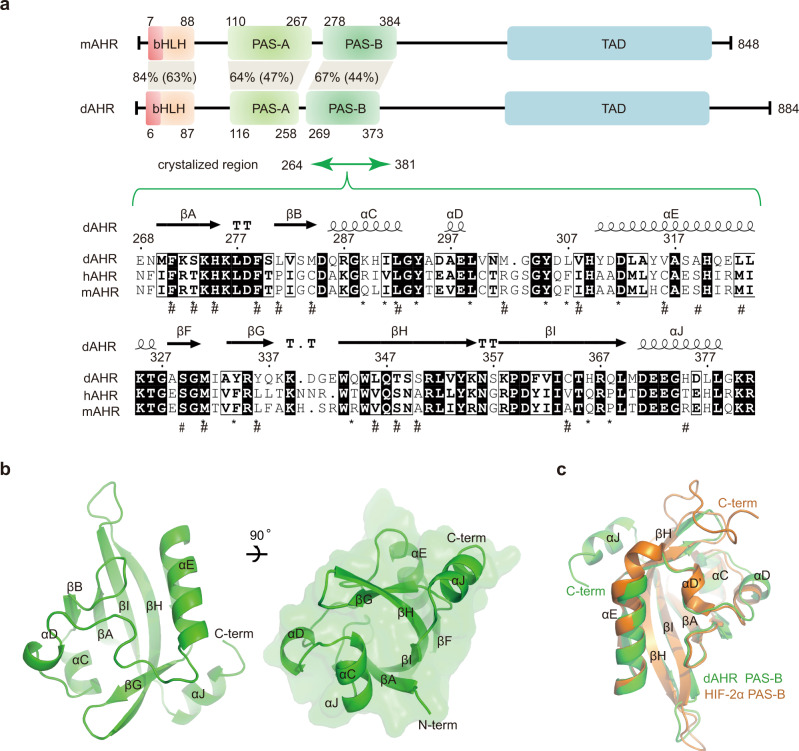
Overall structure of dAHR PAS-B. **a** Domain arrangements of mAHR and dAHR. Amino acid similarities and identities (in parentheses) between the corresponding domains of mAHR and dAHR are indicated. A multiple sequence alignment file of the crystallized sequence is provided. The residues lining the interior cavities of dAHR and mAHR are indicated by ‘#’ and ‘*’, respectively. Sequences were aligned by Clustal Omega and presented by ESPript software. **b** Structure of dAHR PAS-B shown in two views. Secondary structural elements are labeled accordingly. **c** Structural comparison of dAHR PAS-B (green) and HIF-2α PAS-B (colored orange, PDB entry: 6E3U).

AHR belongs to the basic helix-loop-helix/Per-ARNT-SIM (bHLH-PAS) family of transcription factors. From the N- to C-terminus, AHR comprises a basic helix-loop-helix (bHLH) domain for DNA binding, two tandemly arranged PAS domains (termed PAS-A and PAS-B) for dimerization, and a transcription activation domain^12^ (Fig. 1a). Unliganded AHR is localized in the cytosol and forms a protein complex with HSP90, XAP2, P23, and the kinase c-Src^11^. The binding of the AHR agonist induces exposure of its N-terminal nuclear localization sequence (NLS) and leads to translocation of the AHR chaperone complex to the nucleus^11^. Subsequently, AHR is released from the chaperone complex and heterodimerizes with its nuclear partner ARNT. The AHR-ARNT heterodimer recruits a number of transcriptional cofactors^13,14^ and binds the AHR response element (also called the dioxin response element, DRE) to drive the transcription of genes encoding xenobiotic-metabolizing enzymes, such as *CYP1A1* and *CYP1B1*^15^, and immunoregulatory genes, such as *IL-10*^16^.

Among the bHLH-PAS family transcription factors, AHR is the only known member for which activation is ligand-dependent. AHR can bind and be stimulated by a range of structurally divergent ligands exemplified by 2,3,7,8-tetrachlorodibenzo-p-dioxin (TCDD), formylindolo[3,2-b]carbazole (FICZ), indirubin and kynurenines^4,17^. The ligand binding site of AHR has been mapped to its PAS-B domain. Therefore, the AHR PAS-B domain has also been termed the ligand binding domain^18^. The PAS-B ligand binding domain of AHR not only acts as a sensor of environmental and physiological signals but also provides a binding interface for HSP90 and the PAS-B domain of ARNT^19–21^.

Previous studies have provided the structural features of the bHLH and PAS-A domains of AHR. The crystal structure of an isolated mouse AHR (mAHR) PAS-A domain represented the first structure of AHR^22^. A few years later, the structures of the bHLH-PAS-A heterodimer formed between human AHR (hAHR) and mouse ARNT (mARNT) were reported by two separate groups^23,24^. Unlike its bHLH and PAS-A regions, the structure of the AHR PAS-B domain containing the ligand-binding site has not been solved, since the human and mouse PAS-B domains could not be expressed in a soluble form and/or were aggregating^24^. Many efforts have been made to address the structure and ligand-binding characteristics of AHR by using computational modeling in the past two decades^25–29^. These computational-based studies have contributed to our understanding of this functionally essential domain of AHR. However, the lack of an experimental structure has hampered a more detailed and reliable description of AHR ligand binding, as well as the structure-based design of AHR-targeted therapeutics.

In this work, we screen the recombinant expression of over ten AHR PAS-B homologs from different species. Only the PAS-B domain of *Drosophila* AHR (dAHR) is soluble. We determine the crystal structures of dAHR PAS-B domain in multiple forms. By combining biochemical and cell-based functional assays, our study reveals the structural features of the AHR PAS-B domain and provides insights into the mechanism of AHR ligand binding.

## Results

### Structure of the *Drosophila* AHR PAS-B domain

To obtain soluble expressed AHR PAS-B, we screened the recombinant expression of over ten AHR PAS-B homologs from different species. Among them, only the PAS-B domain (residues 264–381) from dAHR was solubly expressed in *E. coli* (Supplementary Fig. 1). dAHR is commonly known as spineless (Uniport entry: O61543), which shares the same domain architecture with mammalian AHR (Fig. 1a)^30^. The bHLH, PAS-A and PAS-B domains of dAHR share 84%, 64% and 67% sequence similarity and 63%, 47% and 44% amino acid identity with the corresponding mAHR regions, respectively (Fig. 1a).

We then performed crystallographic studies on purified dAHR PAS-B. Diffraction-qualified crystals were successfully obtained, and the structure was determined at a resolution of 2.6 Å. The data collection and refinement statistics are summarized in Supplementary Table 1. dAHR PAS-B adopts a typical α/β PAS fold that is shared by other PAS proteins^31^. Topologically, dAHR PAS-B is arranged as βA-βB-αC-αD-αE-βF-βG-βH-βI-αJ and resembles a baseball catcher’s mitt, with the six β-strands forming the palm and three helices (αC, αD and αE) forming the opposing thumb (Fig. 1b).

Next, we compared our dAHR PAS-B structure with the previously reported PAS-B structure of hypoxia-inducible factor-2α (HIF-2α), as the latter is also a bHLH-PAS protein capable of binding ARNT and whose activity can be modulated by synthesized molecules^32–34^. The PAS-B domains of dAHR and HIF-2α share ~47% sequence similarity. Although the overall folding was similar, superimposing the two structures gave a root-mean-square deviation (rmsd) of 4.5 Å over 106 equivalent Cα atoms (Fig. 1c), which indicated comparable differences. The most dramatic differences were observed at the C-terminus. The C-termini adopt opposite conformations in the two structures, with dAHR PAS-B folding into a helix (αJ), while HIF-2α presents as a loop. In addition, part of the residues between αE and αD folded into a short helix (αD’) in HIF-2α, while this helix was not observed in dAHR PAS-B. Other minor differences are observed within the αE, βH, αC and αD regions (Fig. 1c). These observations suggest that dAHR PAS-B has a similar overall fold while adopting some unique features compared to HIF-2α PAS-B.

### Ligand binding properties of dAHR PAS-B

**Fig. 2:**
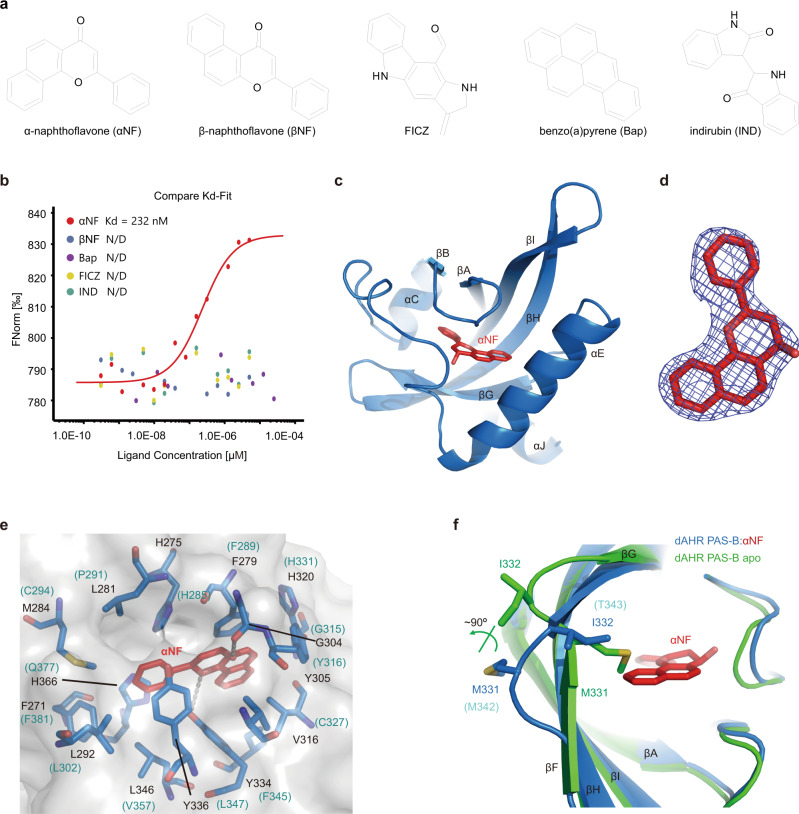
The binding of αNF to dAHR PAS-B. **a** Chemical structure of typical AHR ligands. **b** Binding of different AHR ligands to dAHR PAS-B detected by MST. Data are presented as mean values of two independent experiments. Source data are provided as a Source Data file. **c** Structure of αNF (red sticks) in complex with dAHR PAS-B. **d** The 2Fo-Fc omit map of αNF. The map is shown in gray mesh and contoured at 1.0 σ. **e** The binding of αNF in the dAHR PAS-B internal cavity. Residues shaping the αNF binding site are shown in sticks. The corresponding residues in mAHR PAS-B are shown in parentheses. H-bonds are indicated by dashed lines. **f** Structural comparison of apo dAHR PAS-B (green) and αNF-bound dAHR PAS-B (blue). The side chains of dAHR PAS-B M331 and I332 are shown in sticks. The corresponding mAHR residues M342 and I343 are indicated in parentheses.

It has been demonstrated that dAHR cannot respond to most AHR ligands, such as TCDD and β-naphthoflavone (βNF)^30,35^. The high sequence similarity suggests that dAHR may be capable of binding certain ligands. To test the ligand binding property of dAHR PAS-B, we performed microscale thermophoresis (MST) binding assays. The binding of dAHR PAS-B to five AHR ligands, including βNF, FICZ, indirubin (IND), α-naphthoflavone (αNF), βNF and benzo(a)pyrene (BaP), was tested (Fig. 2a). Among these ligands, only the binding of αNF was detected. The estimated dissociation constant (Kd) of αNF to dAHR PAS-B was approximately 240 nM (Fig. 2b).

Unlike mammalian AHR, dAHR has been found to be constitutively activated in a ligand-independent manner^36,37^. We then employed luciferase reporter gene assays to test whether the presence of αNF could modulate the transcriptional activity of dAHR. However, HEK293T cells overexpressing dAHR did not show significantly increased luciferase levels compared to cells transfected with empty pCDNA3.1(+) plasmid (Supplementary Fig. 2a), suggesting that dAHR has no transcriptional activity in HEK293T cells. This observation is consistent with the previous finding that dAHR may not be activated in mammalian cells^37^.

Next, we constructed a chimeric mAHR protein (mAHR-dPB) with its PAS-B domain replaced by that of dAHR. Compared to wild-type (WT) mAHR, mAHR-dPB has a higher basal activity (Supplementary Fig. 2b). The presence of 1 µM αNF did not alter the activity of mAHR-dPB in HEK293T cells (Supplementary Fig. 2b). In addition, we tested different concentrations of αNF (0.1, 0.5 and 10 µM). No significant change in the luciferase level was observed for mAHR-dPB or dAHR (Supplementary Fig. 2c). Thus, although αNF can bind to dAHR PAS-B, it may not be able to modulate the transcriptional activity of mAHR-dPB in vivo.

### Structure of dAHR PAS-B bound by αNF

To reveal how dAHR PAS-B binds the ligand αNF, we crystallized dAHR PAS-B in the presence of this chemical. The complex crystals were grown under the same conditions as the apo protein, and the structure was determined to a resolution of 2.4 Å (Fig. 2c and Supplementary Table 1). αNF lies in the middle of the dAHR PAS-B cavity with a well-defined electron density (Fig. 2d) and forms extensive hydrophobic interactions with dAHR PAS-B (Fig. 2e and Supplementary Fig. 3a). In addition, two H-bonds contributed by the Y334 side chain and the G304 carbonyl oxygen of dAHR PAS-B were also observed (Fig. 2e and Supplementary Fig. 3a). Notably, the corresponding Y334 in dAHR is replaced by F345 in mAHR (Fig. 2d); therefore, the H-bond contributed by dAHR PAS-B Y334 may not be expected when αNF binds to mAHR.

Compared to apo dAHR PAS-B, αNF-bound dAHR PAS-B undergoes a dramatic conformational change within its βG-βF region. Residues M331 and I332 undergo an approximately 90° rotation, resulting in the side chain of M331 flipping away from the cavity to facilitate αNF insertion (Fig. 2f). As this methionine is also conserved in mAHR (M342) (Fig. 2f), a similar binding-induced conformational change may occur when it is bound by the same ligand. We then constructed the mAHR M342A mutant and performed DRE luciferase reporter assay to evaluate the biological relevance of αNF binding-induced conformational change. Compared to WT mAHR, M342A had similar levels of transcriptional activity in the presence or absence of different ligands (Supplementary Fig. 3b). These observations suggest that the conformational change induced by the binding of αNF may not be directly involved in the regulation of mAHR transcriptional activity.

### Analysis of the differences in ligand binding between dAHR and mAHR

Our MST binding analysis showed that dAHR could not bind most AHR ligands. To uncover the underlying reason, we simulated the binding of different AHR ligands to mAHR PAS-B by computational docking. The structure mode of mAHR PAS-B was generated by Modeller^38^ with our determined dAHR PAS-B structure used as the single homology template. Our docking results are very similar to those of a previous docking study that used a different docking methodology^29^, suggesting that the docking results are reliable.

**Fig. 3:**
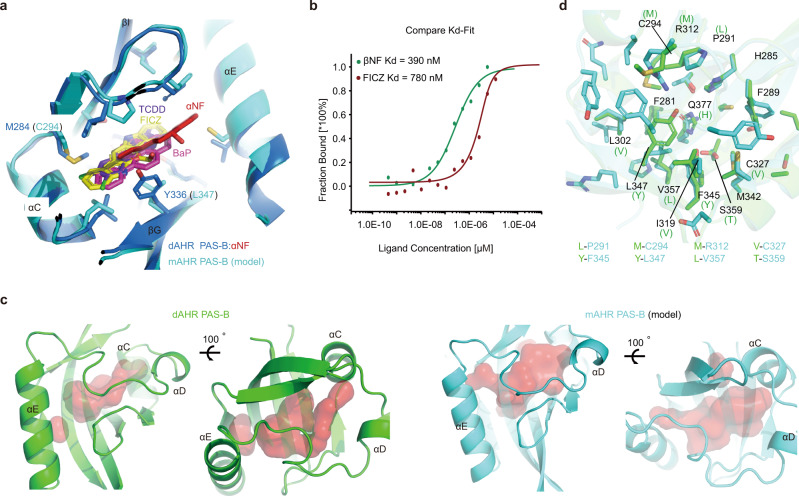
Comparison of the ligand-binding cavities of dAHR PAS-B and mAHR PAS-B. **a** Superposition of the dAHR PAS-B:αNF complex (blue) and mAHR PAS-B:ligand (cyan) docking models. The mAHR PAS-B structure model was created by the program Modeller with the apo dAHR PAS-B structure used as the single template. Ligands are shown in sticks. **b** Ligand binding property of the dAHR PAS-B M284C/Y336L mutant measured by MST. Data are presented as mean values of two independent experiments. Source data are provided as a Source Data file. **c** The relative position of the ligand-binding cavity (red surface) of dAHR PAS-B and mAHR PAS-B. Cavity sizes were calculated by the program CASTp using the default probe sphere radius of 1.4 Å. **d** Cavity comparison of dAHR PAS-B and mAHR PAS-B. Nonconserved residues in the dAHR PAS-B domain are shown in parentheses. A list of mAHR PAS-B residues that evolved from large to small side chains is listed at the bottom.

The predicted binding sites of TCDD, BaP and FICZ are at the bottom of the cavity (βB, αC, αD region) (Fig. 3a). The superposition of the dAHR PAS-B:αNF structure with the computational docking results showed that the binding positions of the docked ligands are much deeper in the pocket (Fig. 3a). For dAHR PAS-B, the binding of these three docked agonists may be hindered by residues Y336 and M284. In contrast, the corresponding residues are replaced by L347 and C294 in mAHR, and both residues are equipped with a smaller side chain. We then constructed the dAHR PAS-B variant bearing the M284C/Y336L mutation and evaluated its ligand-binding ability by MST. Interestingly, M284C/Y336L mutant gained the ability to bind βNF and FICZ with Kd values of approximately 400 nM and 800 nM, respectively (Fig. 3b). These results suggest that by modifying its ligand-binding pocket, dAHR PAS-B can obtain the ability to bind different AHR ligands.

Furthermore, we compared the internal cavities of dAHR PAS-B and mAHR PAS-B (homology model). CASTp^39^ assigns 440 Å^3^ and 680 Å^3^ cavity volumes for dAHR PAS-B and mAHR PAS-B, respectively (Fig. 3c). Compared to dAHR PAS-B, the cavity of mAHR PAS-B is expanded in all three dimensions. Although their sizes are different, dAHR and mAHR PAS-B domains utilize almost the same constellation of amino acids to form ligand cavities (Fig. 3c). Most of the lining residues in mAHR have been substituted by residues with a smaller side chain than dAHR PAS-B (Fig. 3d). The observed variations in the mAHR PAS-B cavity are consistent with its broad adaptability to chemicals with different structures and sizes, whereas the ligand-binding ability of dAHR may be reduced by its limited cavity size.

### dAHR PAS-B binds mouse ARNT PAS-B directly

**Fig. 4:**
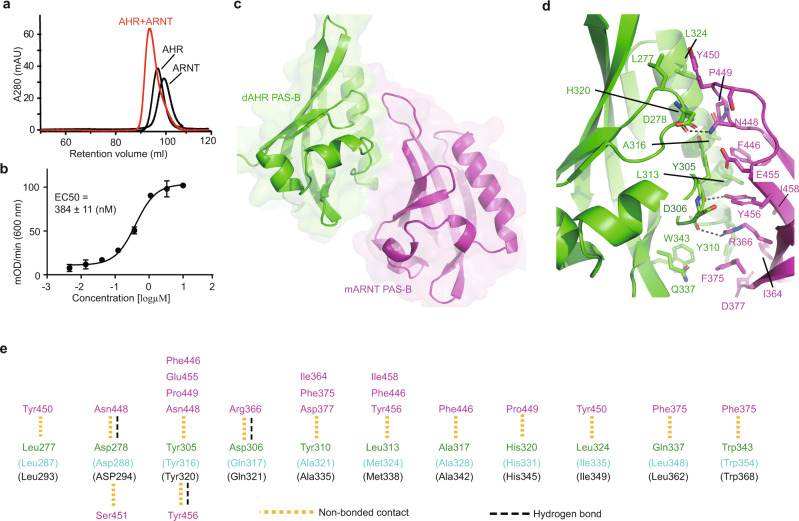
Structure of the dAHR PAS-B:mARNT heterodimer. **a** Evaluation of the interaction between dAHR PAS-B and mAHR PAS-B by SEC. **b** The binding affinity of dAHR PAS-B to mARNT PAS-B measured by ELISA. The results represent the average value of three independent experiments. Data are shown the mean ± standard deviation of *n* = 3 independent replicates. Source data are provided as a Source Data file. **c** The overall structure of the dAHR PAS-B:mARNT heterodimer. dAHR PAS-B is colored in green cartoon and mARNT PAS-B in magenta. **d** Detailed interactions at the dimer interface. Interaction residues are shown in sticks. H-bonds are indicated by dashed lines. **e** Schematic diagram of the detailed contacts at the PAS-B heterodimer interface. The corresponding mAHR (cyan) and hAHR (black) PAS-B residues are indicated in parentheses.

To be transcriptionally activated, dAHR must form a heterodimer with tango, the *Drosophila* ARNT homolog^30,40^. mAHR has been shown to bind tango in *Drosophila* to regulate gene transcription^41^. Hence, we speculated that dAHR could also bind mouse ARNT (mARNT). More specifically, dAHR PAS-B may physically bind mARNT PAS-B. To test this hypothesis, we purified the mARNT PAS-B domain and evaluated the interaction by size exclusion chromatography (SEC). The retention volumes of dAHR PAS-B and mARNT PAS-B were 96.7 and 99.2 ml on a HiLoad 16/60 Superdex 200 column, respectively. The elution of dAHR PAS-B occurred slightly earlier than that of mARNT PAS-B, which is consistent with the sizes of these two proteins (dAHR PAS-B 13.6 kDa, mARNT PAS-B 12.6 kDa) (Fig. 4a). The loading of the dAHR PAS-B and mARNT PAS-B mixture resulted in a single elution peak that appeared ahead of the dAHR PAS-B protein alone, indicating that dAHR PAS-B and mARNT PAS-B formed a complex in solution.

Furthermore, we purified GST-tagged mARNT PAS-B and 6xHis-SUMO-tagged dAHR PAS-B and employed enzyme-linked immunosorbent assay (ELISA) to quantitate their binding affinity. The measured binding affinity between mARNT PAS-B and dAHR PAS-B was approximately 380 nM (Fig. 4b). Our SEC analysis and ELISA demonstrated that dAHR PAS-B could bind mARNT PAS-B directly.

### Structure of the dAHR:mARNT PAS-B heterodimer

To characterize how dAHR PAS-B binds mARNT, we determined the crystal structure of dAHR PAS-B in complex with mARNT PAS-B (Supplementary Fig. 4a and Table 1). The crystal asymmetric unit (ASU) contains two dAHR PAS-B and two mARNT PAS-B molecules, which form two closely related heterodimers with a rmsd of 0.34 Å over 212 aligned Cα atoms (Supplementary Fig. 4b). In the heterodimer, the two PAS-B molecules are organized in a roughly parallel orientation with a total buried surface of 1160 Å^2^ (Fig. 4c). dAHR PAS-B:mARNT dimerization is predominantly mediated by hydrophobic interactions. Three hydrophobic residues, Y305, Y310 and L313, of dAHR PAS-B interact most extensively with mARNT PAS-B (Fig. 4d, e). The side chains of R366, N448 and Y456 of mARNT PAS-B form hydrogen bonds (H-bonds) with dAHR PAS-B W343, D278 and D306, respectively. Interestingly, all the observed H-bonds are facilitated by carbonyl oxygens rather than the side-chain atoms of dAHR PAS-B. Of all 11 dAHR PAS-B residues participating in the interaction with mARNT, 9 are conserved in mAHR and hAHR (Fig. 4e), suggesting that the AHR-ARNT PAS-B interaction pattern may be highly conserved among these species.

Next, we compared the structure of mARNT-bound dAHR PAS-B with its apo form. The superimposition of apo dAHR PAS-B on the heterodimer gave a rmsd of 0.78 Å, indicating that no significant conformational change appeared. Some modest conformational differences were observed in the αD, αE, βH and βI regions (Supplementary Fig. 4c). However, these differences may be caused by model bias, resolution limitations, or different crystal packing environments.

### Mutational analysis of the dimer interface

**Fig. 5:**
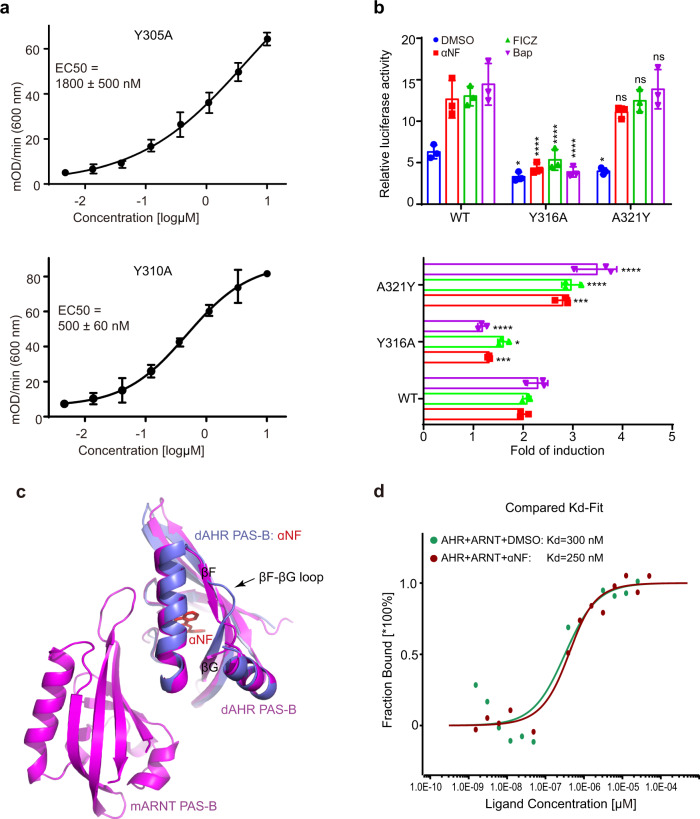
Analysis of the dAHR:mARNT PAS-B heterodimer interface. **a** The binding affinities of dAHR PAS-B mutants to mARNT PAS-B measured by ELISA. **b** DRE luciferase reporter assay examining the effect of AHR mutation on the transactivation of mAHR in HEK293T cells in the presence or absence of agonists. FICZ, βNF and BaP were supplied at 200 nM, 500 nM and 200 nM, respectively. The lower panel shows the fold of induction in luciferase activity due to ligand treatment (luciferase activity in the presence of ligand divided by that in the presence of DMSO). **c** Superimposition of the dAHR PAS-B:αNF and dAHR PAS-B:mARNT complex structures. **d** Analysis of the impact of αNF on the interaction between dAHR PAS-B and ARNT. Data for **d** are the mean of two independent experiments, and data for (**a**, **b**) are the mean ± standard deviation of *n* = 3 independent replicates. *p* values were calculated using two-way ANOVA with Tukey’s multiple comparisons test with the WT mAHR group treated by the same ligand as a control and presented in the Source Data file. *p < 0.05, ***p < 0.001, ****p < 0.0001, ns: not statistically significant (*p* > 0.05). Source data are provided as a Source Data file.

The AHR-ARNT heterodimer structure showed that Y305 and Y310 of dAHR PAS-B form extensive hydrophobic interactions with mARNT PAS-B. To evaluate the contribution of these two tyrosine residues to heterodimerization, we constructed and purified dAHR PAS-B Y305A and Y310A mutants and quantified their ARNT-binding affinities by ELISA. Compared to the WT protein (380 nM), the binding strength of Y305A to mARNT was largely decreased (1800 nM), while it was only slightly decreased for Y310A (500 nM) (Fig. 5a). These observations suggest that dAHR Y305 plays a key role in mediating its dimerization with the nuclear partner.

Sequence alignment showed that Y305 of dAHR is conserved in mAHR (Y316), whereas Y310 is substituted by alanine (A321) in mAHR (Fig. 1a). To assess the role of Y316 and A321 in the transcriptional activity of mAHR, we constructed two mAHR mutants, Y316A and A321Y, and performed DRE luciferase reporter assays (Fig. 5b). The mutation of Y316 to alanine caused a significant decrease in luciferase activity compared to the WT mAHR both in the absence and presence of AHR agonists. Like Y316A, A321Y basal activity was significantly decreased compared to WT mAHR. However, its activities induced by different agonists were at a similar level as the WT mAHR (Fig. 5b). When comparing the fold increase in luciferase activity due to ligand treatment (ligand treatment divided by DMSO treatment), Y316A showed a reduced, while A321Y showed an increased induction levels (Fig. 5b, lower panel). These results are consistent with the structural observations that the highly conserved tyrosine of AHR (Y305 in dAHR, Y316 in mAHR) plays a key role in mediating its dimerization with ARNT, and the mutation of mAHR A321 to tyrosine could enhance its interaction with ARNT.

### αNF binding does not affect dAHR PAS-B:mARNT interaction

The crystal structure showed that the binding of αNF induces a large conformational change in the βF-βG region of dAHR PAS-B. The superimposition of the αNF-bound dAHR PAS-B and the AHR:ARNT heterodimer showed that αNF binding-induced conformational change occurred outside the heterodimer interface (Fig. 5c), indicating that the binding of αNF may not affect the interaction between dAHR PAS-B and mARNT PAS-B. To test this hypothesis, we measured the binding affinities of dAHR PAS-B to ARNT in the presence and absence of αNF by MST (Fig. 5d). In the absence of αNF, MST assigned the dAHR PAS-B:mARNT interaction a binding constant (Kd) of 300 nM, which is similar to that quantified by ELISA (380 nM). Similar to the binding in the absence of αNF, dAHR PAS-B binds to ARNT at a Kd of approximately 250 nM in the presence of this compound. Therefore, the binding of αNF does not directly affect the interaction between dAHR PAS-B and mARNT PAS-B.

### Structural comparison with other PAS-B heterodimers

**Fig. 6:**
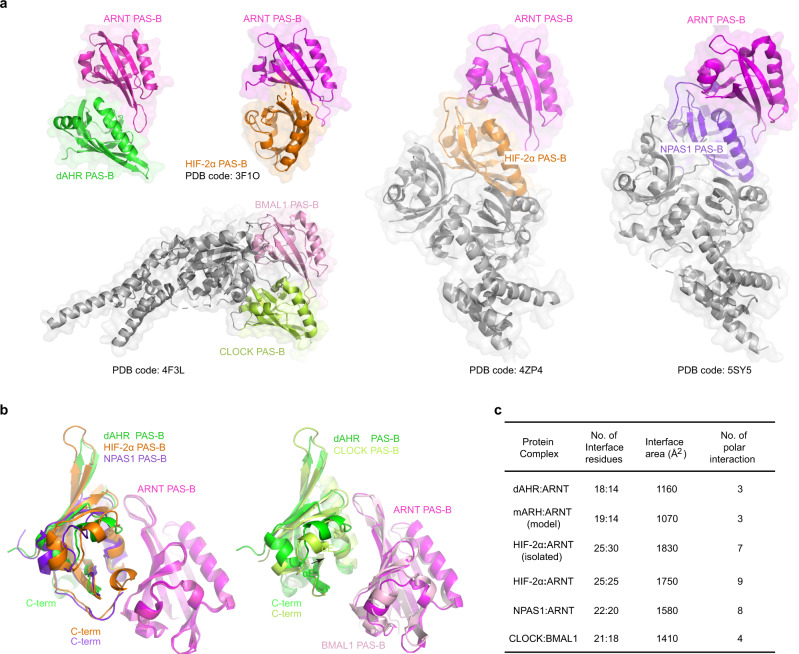
Comparison of the interaction interfaces of different PAS-B heterodimers. **a** PAS-B:PAS-B interaction interfaces observed in different bHLH-PAS heterodimers. The PAS-B domains are highlighted, while other domains (bHLH and PAS-A) are shown in gray. **b** Superimposition of the PAS-B heterodimers. For the CLOCK:BMAL1, HIF-2α:ARNT and NPAS1:ARNT complexes, only the PAS-B regions were present. **c** A table summarizing the interface features for each PAS-B heterodimer. The parameters are calculated by PISA^60^. Polar interactions, including H-bonds and salt bridges, are defined at a distance cutoff of 3.5 Å.

We then compared our dAHR PAS-B:ARNT structure with other PAS-B heterodimers, including HIF-1α/HIF-2α:ARNT^42^, NPAS1/NPAS3:ARNT^43^, and CLOCK:BMAL1^44^ complex structures (Fig. 6a). These PAS-B heterodimers share a similar dimerization pattern, with the β-sheet of one PAS-B molecule serving as a binding platform and touched by the helical bundle from the other molecule. Interestingly, a different interface has been observed in isolated HIF-1α/HIF-2α:ARNT PAS-B heterodimer structures, where the two PAS-B subunits are arranged in an antiparallel orientation with the respective β-sheet mediating dimerization^45,46^ (Fig. 6a).

The superimpositions of dAHR PAS-B:ARNT with HIF-2α:ARNT, NPAS1:ARNT and CLOCK:BMAL1 gave rmsd values of 1.4, 1.3 and 5.8 Å, respectively (Fig. 6b), indicating that the overall structure of the dAHR PAS-B:ARNT PAS-B complex is very similar to that of HIF-2α:ARNT and NPAS1:ARNT. Compared to HIF-2α:ARNT (1750 Å^2^) and NPAS1:ARNT (1580 Å^2^), the size of the dAHR PAS-B:ARNT dimerization interface area is much smaller (1160 Å^2^) (Fig. 6b, c). The C-terminal tails of HIF-2α and NPAS1 PAS-B wrapped toward the interface, making additional contacts with ARNT PAS-B, while the C-termini of dAHR PAS-B pointed in the opposite direction (Fig. 6b). Accordingly, electrostatic interactions and the number of interface residues are decreased in the AHR:ARNT PAS-B heterodimer. Notably, the C-terminus of CLOCK PAS-B adopts a similar orientation as that of dAHR PAS-B, and its larger-size interaction interface (1410 Å^2^) may predominantly be achieved by shifting its αE helix toward BMAL1 (Fig. 6b).

Furthermore, we made a mAHR:mARNT PAS-B heterodimer model by roughly overlaying the predicted mAHR PAS-B structure onto the dAHR PAS-B:ARNT structure (Supplementary Fig. 5). The obtained heterodimer model is almost identical to the previous models built based on the HIF-2α:ARNT and CLOCK:BMAL1 structures^47,48^. Compared to dAHR:mARNT PAS-B heterodimer (1160 Å^2^), the mAHR:mARNT PAS-B heterodimer possesses an even smaller interface (1070 Å^2^). The underlying reason why AHR:ARNT prefers a small PAS-B interface is not clear, and we speculate that a weak dimerization interface may allow the regulation of AHR activity to be more flexible.

## Discussion

AHR is an ancient cellular sensor under approximately 600 million years of evolution and is widely distributed in the animal kingdom^49^. Previous studies have shown that, unlike vertebrate AHR, invertebrate AHR orthologs do not appear to act as sensors and cannot be stimulated by typical AHR ligands^36,37^. It has been demonstrated recently that the transcriptional activity of *Caenorhabditis elegans* (*C. elegans*) AHR-1 (cAHR-1) could be modulated by some AHR ligands, indicating that it may be directly bound by those ligands^50^. Here, we also showed that the dAHR PAS-B domain could bind directly to αNF. It is likely that the ligand-binding ability of AHR is an ancestral trait. Although dAHR PAS-B could be bound by αNF, as inferred by the chimeric mAHR-dPB, the binding of αNF may not be able to modulate its transcription activity. One possible explanation is that the ligand simply acts as a switch for nuclear translocation of the AHR, and once translocated, the transcriptional activity of AHR is independent of the bound ligand.

Although it has long been characterized as a ligand-regulated transcription factor, the molecular mechanism of AHR ligand binding and how binding ligands modulate its activity are unclear. Here, we showed that αNF binds to the dAHR PAS-B domain at a pocket near αE, βH, βG and αD’. A similar ligand binding position has been observed for other PAS proteins, such as human HIF-2α^34,51^ and *E. coli* Dos^52^ (Supplementary Fig. 7). Thus, the binding of αNF in dAHR PAS-B is at a canonical position in the pocket of the PAS domain. Ligand binding assays of HIF-2α showed that the binding of the antagonist displaces HIF-2α M252 away from the cavity to the HIF-2α:ARNT heterodimer interface. This conformational change destabilized the heterodimer and resulted in decreased HIF-2α transcriptional activity. In contrast, the binding of the agonist displaces HIF-2α Y281 from the inside cavity to further stabilize the heterodimer^34^. Our structure showed that AHR and HIF-2α PAS-Bs utilized the same interface to bind ARNT. However, HIF-2α M252 and Y281 are not conserved in AHR, and αNF-binding-induced conformational changes occur outside the AHR:ARNT PAS-B heterodimer interface. In addition, our luciferase reporter gene assays indicated that disturbing the αNF binding-induced conformational change may not affect mAHR transcriptional activity. Taken together, these observations further support our hypothesis that AHR activity may not be modulated by the bound ligand after its nuclear translocation.

Molecular docking indicated that C294 and L347 of mAHR are important in permitting its ligand binding. Multiple sequence alignment showed that these two residues are highly conserved in vertebrate AHRs (for example, human, mouse, zebrafish and *Xenopus laevis*) but not fully conserved in *Drosophila* or *C. elegans* (Supplementary Fig. 6). The corresponding residues in dAHR are substituted by a methionine (M284) and tyrosine (Y336) in dAHR. We showed that when these two residues are mutated to the corresponding mAHR residues, dAHR acquired the ability to bind different ligands (Fig. 3b). Although these two residues are not fully conserved in cAHR-1, the transcriptional activity of cAHR-1 has recently been shown to be modulated by several AHR ligands^50^. Unlike dAHR, cAHR-1 PAS-B possesses a leucine instead of a tyrosine at its corresponding position (Supplementary Fig. 6). These observations indicate that the conserved leucine may play a crucial role in determining the ligand binding diversity of AHR.

Of all 12 tested AHR PAS-B domains, only dAHR PAS-B was recombinantly expressed in a soluble and homogeneous state. Subsequent crystal structure analysis showed that dAHR PAS-B adopts a typical PAS fold, although it was suggested that AHR PAS-B might adopt a different fold than other PAS proteins^53^. Combined with the fact that the activation of dAHR is constitutive and ligand-independent, we infer that dAHR PAS-B might be in an intrinsically active conformation. In contrast, the folding of other AHR PAS-B domains into the typical PAS architecture may require the binding of a ligand or the involvement of other regulatory mechanisms. As indirect evidence, AHR is the only known bHLH-PAS protein required to be bound by the chaperone HSP90 in the cytoplasm.

In summary, we successfully expressed and purified dAHR PAS-B and solved the structure of the AHR PAS-B domain. We also determined the crystal structure of the dAHR PAS-B:αNF complex and provided direct observation of ligand-specific AHR structural changes. In addition, we solved the crystal structure of the heterodimer formed by the PAS-B domains from dAHR and mouse mARNT. Together with biochemical and cellular assays, our data provide structural insights into the mechanism of AHR ligand binding and should assist future efforts in rational development of therapeutics targeting AHR.

## Methods

### Plasmid construction and site-directed mutagenesis

DNA-encoding sequences of AHR PAS-B domain from different species, including *Homo sapiens* (Uniport entry: P35869 and here after, aa 280–398), *Mus musculus* (P30561, aa 274–392), *Oryctolagus cuniculus* (O02747, aa 278–396), *Danio rerio* (Q9YGV3, aa 285–403), *Drosophila melanogaster* (O61543, aa 264–381), *Gallus gallus* (F1NLX8, aa 279–397), *Canis lupus familiaris* (A0A8I3RSX6, aa 263–381), *C. elegans* (O44712, aa 280–397), *Bos taurus* (F1ML85, aa 279–397), *Xenopus laevis* (B7ZS97, aa 269–387), *Equus caballus* (F6ZNC3, aa 279–397) and *Sus scrofa* (I3LF82, aa 278–396), were synthesized and subcloned into a modified pET-28a vector (pET-28s). The DNA encoding the mARNT PAS-B domain (aa 360–465) was synthesized and subcloned into the pET-28s plasmid similarly.

The mARNT PAS-B-encoding sequence was also subcloned into pGEX-6P-1 to express GST-tagged recombinant protein for enzyme-linked immunosorbent assay (ELISA). Full-length mAHR and dAHR were cloned into the pCDNA3.1(+) vector for transactivation assays. The chimeric mAHR-dPB construct was generated by replacing the mAHR 274–392 coding sequence with that of dAHR 264–381 through homologous recombination technology (Vazyme, ClonExpress II One Step Cloning Kit). All site-directed mutants were obtained with the KOD-Plus-Mutagenesis kit (TOYOBO, SMK-101) and confirmed by DNA sequencing.

### Protein expression and purification

The recombinant plasmids pET-28s-dAHR PAS-B and pET-28s-mARNT PAS-B were transformed into *E. coli* strain Rosetta (DE3) cells for expression. The cells were grown at 37 °C and induced with 0.2 mM IPTG when the culture OD_600_ reached 0.8 and further incubated for 12 h at 20 °C. Cells were harvested by centrifugation. For purification, cells were resuspended and lysed in a lysis buffer containing 20 mM Tris-HCl pH 8.0, 500 mM NaCl, and 3 mM β-ME2 with a high-pressure homogenizer. The cell lysate was further clarified by centrifugation at 18,000 rpm for 30 min. Then, the supernatant was subjected to nickel-affinity chromatography and subsequently treated with ULP1 protease to remove the fusion tag. After overnight digestion, the imidazole was removed by buffer change with a centrifugal filter unit (Millipore) and then reloaded to a Ni-NTA column to remove the tag, followed by size exclusion chromatography (SEC) with 20 mM Tris-HCl pH 8.0, 150 mM NaCl, and 0.5 mM TCEP used as the running buffer. Purified proteins (dAHR PAS-B and mARNT PAS-B) were concentrated to 0.9 mM, flash-frozen in liquid nitrogen, and stored at −80 °C for further usage.

The purification of dAHR PAS-B mutants followed the same steps as described for the WT protein. For ELISA, the 6xHis-SUMO fusion tag (dAHR PAS-B) and GST fusion tag (mARNT PAS-B) were retained.

### Crystallization

The PAS-B heterodimer complex was prepared by mixing dAHR PAS-B and mARNT PAS-B at a molar ratio of 1:1 (with a final complex concentration of 0.5 mM) and incubated on ice for 1 h before crystallization. dAHR PAS-B and the heterodimer were screened for crystallization conditions with the help of a Phenix crystallization robot (Art Robbins Instruments) using the sitting drop vapor-diffusing technique in a 96-well plate. For each condition, 0.2 µl of protein (0.9 mM for dAHR PAS-B and 0.45 mM for the heterodimer) and 0.2 µl of reservoir solution were mixed; the mixture was equilibrated against 60 µl of the reservoir at 18 °C. The crystal of dAHR PAS-B appeared under the condition of 0.1 M Tris-HCl pH 7.0, 0.2 M NaCl, 0.8 M sodium citrate. The crystallization condition for the PAS-B heterodimer is 0.1 M sodium arsenate (pH 6.5), 0.2 M MgCl_2_, 1.2 M (NH_4_)_2_SO_4_.

The dAHR PAS-B:αNF complex was prepared by mixing dAHR PAS-B and αNF stock solution (30 mM) in a molar ratio of 1:1.2. The crystals were grown under the same conditions as apo dAHR PAS-B. All crystals were treated with cryoprotectant buffer consisting of the corresponding reservoir solution plus 20% (v/v) glycerol and flash-frozen in liquid nitrogen prior to data collection.

### X-ray diffraction data collection and structure determination

The X-ray diffraction data set of apo dAHR PAS-B was collected using an in-house MicroMax-007 X-ray generator equipped with VariMax HR optics (Rigaku, Japan). The data sets of the dAHR PAS-B:αNF and dAHR PAS-B:ARNT complexes were collected at the BL17U1 beamline of the Shanghai Synchrotron Radiation Facility (SSRF). All data were indexed and processed with the program suite HKL2000^54^. The structure of dAHR PAS-B was determined by MR with the program phenix.phaser^55^, with the human HIF-2α PAS-B structure (PDB entry: 3F1O) used as the search model. The initial model was rebuilt manually in Coot^56^. Refinements were performed with Phenix.refine^57^. The final model was completed after alternative rounds of model building and refinement. The αNF-bound dAHR PAS-B structure was resolved by MR using apo dAHR PAS-B as the search model. The refined apo dAHR PAS-B and the mouse ARNT PAS-B structures (PDB entry: 3F1O) served as two separate search models to determine the heterodimer complex structure by MR. The statistics of data collection and structure refinement are summarized in Table S1. All structural figures were prepared using PyMOL.

### MST binding assay

MST binding assays were carried out on a Monolith LabelFree instrument (NanoTemper Technology). AHR ligands were diluted in a range of concentrations (from 5 µM to 0.15 nM) and mixed with 350 nM purified dAHR PAS-B at room temperature in buffer containing 20 mM Tris pH 8.0, 100 mM NaCl, 0.5 mM TCEP, 2.5% DMSO and 0.1% F-127. For the AHR-ARNT interaction assay, purified 6xHis-tagged dAHR PAS-B was labeled with the MO Red-Tris-NTA protein labeling kit. mARNT PAS-B was serially diluted to concentrations from 100 µM to 3 nM and mixed with 50 nM Red-labeled dAHR PAS-B in the presence or absence of 1 µM αNF in PBS buffer containing 0.05% Tween−20, 2.5% DMSO and 0.5 mM TCEP. Thermophoresis data were recorded at the default parameters provided by MO. Control program. Kd values were obtained by fitting the MST data using MO. Affinity Assay software.

### Luciferase reporter assay

HEK293T cells were grown in Dulbecco’s modified Eagle medium (DMEM) with 10% fetal bovine serum (FBS) and 1% penicillin–streptomycin. Cells were seeded in 96-well plates overnight and transfected with 50 ng pCDN3.1-mAHR (full-length WT, mutants or empty plasmid), 100 ng pGL3-DRE-promoter and 20 ng pRL-TK (control Renilla luciferase) using 0.5 µl PEI 25k for each well. 20 h after transfection, cells were treated with the corresponding ligands (FICZ, 200 nM; βNF, 500 nM; BaP, 200 nM; αNF, 1 µM; CH-223191, 1 µM) or DMSO (final concentration of 0.1%) and further cultured for 10 h. Luciferase activity was measured on a multi-mode plate reader (Perkin-Elmer) by using a dual luciferase reporter gene assay kit (Beyotime) and EnVision Manager software. Data were normalized to the relative ratio of firefly and Renilla luciferase activity. The experiments were performed in triplicate and repeated three times.

### Size exclusion chromatography

SEC assays were carried out at 4 °C on an ÄKTA Explorer system using a HiLoad 16/60 Superdex 200 prep grade column (Ge healthcare). All chromatography runs were performed using 20 mM Tris-HCl, pH 8.0, 250 mM NaCl, and 2 mM DTT as the running buffer at a flow rate of 1 ml/min. The chromatographic profiles of dAHR PAS-B and mARNT PAS-B were measured by loading 800 µl of the sample at a concentration of 60 µM onto the column. To measure the dAHR PAS-B (WT or mutants):ARNT mixture profiles, samples were prepared by mixing dAHR PAS-B and mARNT PAS-B at a molar ratio of 1:1 with a final complex concentration of 60 µM and injected in the same way as the individual PAS-B proteins. Data were processed and presented using the program Origin8.

### ELISA

ELISAs were carried out with the GST 6xHis-tag ELISA kit (Abcam, ab128573) according to the user manual. Briefly, 50 µl of 100 nM 6xHis-SUMO-dAHR PAS-B protein (WT or mutants) was coated onto 96-well plates for 2 h at room temperature. After coating, the plates were washed 3 times with washing buffer (provided in the assay kit). 15 µl of serially diluted GST-fused recombinant mARNT PAS-B (from 0.004 µM to 10 µM) was added and incubated for 1 h at room temperature. Each well was aspirated and washed three times, and 50 µl of prepared primary detector antibody (anti-GST antibody, provided in the kit, 10-fold diluted) was added to the wells and incubated for 1 h at room temperature. Each well was aspirated and washed three times. Then, 50 µl HRP-labeled secondary detector antibody (provided in the kit, 10-fold diluted) was added and incubated for 1 h at room temperature. After three aspirations and washes, we added 100 µl HRP Development Solution to each well. The assays were quantified by reading the absorbance at 600 nm with a multi-well plate reader. The curves were fitted using the “log(agonist) vs response—Variable slope (four parameters)” function in Prism 7 (GraphPad Software).

### Molecular docking

Molecular docking was performed by AutoDock 4.2 software^58^. The structures of TCDD, FICZ and BaP were drawn in ChemDaw and converted to their corresponding three–dimensional coordinates with eBLOW in the PHENIX software suite^59^. Hydrogen atoms and Gasteiger charges were assigned by AutoDock Tools (ADT). All ligand torsion angles were detected to enable flexible docking. The mAHR PAS-B homology model was built by Modeller^38^ with the apo dAHR PAS-B structure used as the single template.

The standard procedure was adopted for rigid protein and flexible ligand docking. One unique grid box is centered on the center of mAHR PAS-B with dimensions of 60 × 50 × 50 Å^3^, which encompasses the entire ligand-binding cavity. A total of 150 cycles of Lamarckian Genetics Algorithm calculations were carried out to perform the ligand conformational search, and 200 conformations were generated for each ligand. All conformations were clustered according to rmsd within 0.5 Å. The conformation with the lowest docking energy in the most populated cluster was finally selected.

### Reporting summary

Further information on research design is available in the Nature Research Reporting Summary linked to this article.

## Supplementary information

Supplementary Information

Reporting Summary

Peer Review File

## Data Availability

Structure coordinates and map files generated in this study are deposited into the RSCB Protein Data Bank (PDB) with the following accession codes: PDB ID 7VNA (apo dAHR PAS-B), PDB ID 7VNH (αNF-bound dAHR PAS-B), and PDB ID 7VNI (heterodimer). Structure coordinates used in this study are accessible in PDB under the following accession codes: 3F1O, 6E3U, 4ZP4, 4F3L, 5SY5, 3H82, 1V9Z. Source data are provided with this paper as a source data file. Source data are provided with this paper.

## Source data

Source Data
