# Supplementary material for: Structural insight into the ligand binding mechanism of aryl hydrocarbon receptor
Source: Nat Commun. 2022 Oct 20;13:6234. doi: 10.1038/s41467-022-33858-w (PMC9585082; doi:10.1038/s41467-022-33858-w)
Supplement: Supplementary file 1 — Supplementary Information [file 41467_2022_33858_MOESM1_ESM.pdf]

**Supplementary Information**

# Structural insight into the ligand binding mechanism of aryl hydrocarbon receptor

Shuyan Dai[1], Lingzhi Qu[1], Jun Li[2], Ye Zhang[1], Longying Jiang[1], Hudie Wei[1], Ming Guo[1], Xiaojuan Chen[1], Yongheng Chen[1*]

1 Department of Oncology, NHC Key Laboratory of Cancer Proteomics & State Local Joint Engineering Laboratory for Anticancer Drugs, National Clinical Research Center for Geriatric Disorders, Xiangya Hospital, Central South University, Changsha, Hunan, 410008, China

2 Institute of Clinical Medicine, The First Affiliated Hospital of University of South China, Hengyang, Hunan 421001, China

*Correspondence, yonghenc@163.com (Y.C.)

## Content:

**Supplementary Fig. 1-7**

**Supplementary Table 1**

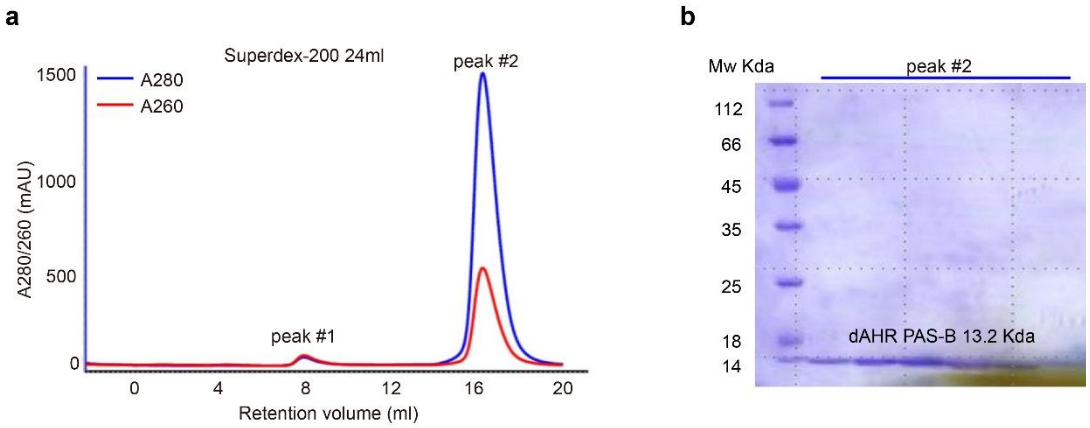


**Supplementary Fig. 1 Recombinant expression and purification of dAHR PAS-B domain. a** SEC profile of purified dAHR PAS-B. **b** SDS-PAGE analysis of dAHR PAS-B. These results are representative of at least three independent experiments. Source data are provided as a Source Data file.

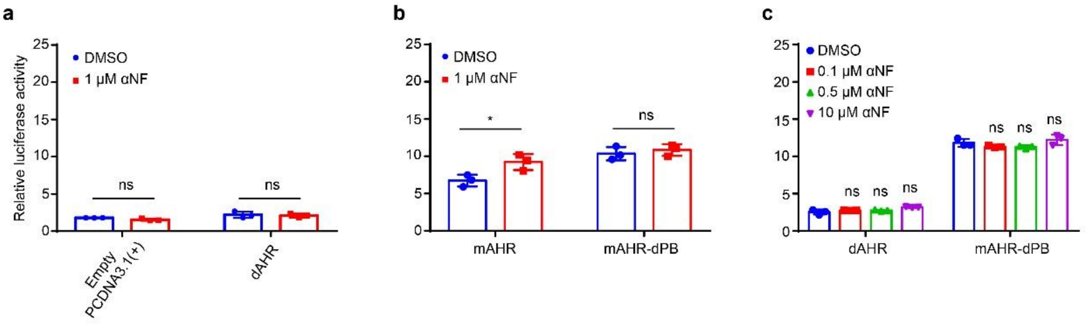


**Supplementary Fig. 2 Luciferase gene reporter assays performed for dAHR and mAHR-dPB. a** dAHR shows little or no transcriptional activity in HEK293T cells. **b** mAHR-dPB has a higher basal activity than mAHR, and the presence of αNF does not impact its transcriptional activation. **c** Different concentrations of aNF have no impact on the activity of mAHR-dPB or dAHR. n = 3 biological repeats for each group. Data are shown as mean ± SD. *p*-values were calculated using a two-way ANOVA with Tukey's multiple comparisons test and presented in the Source Data file. *: $p<0.05$, ns: not statistically significant ($p > 0.05$). Source data are provided as a Source Data file.

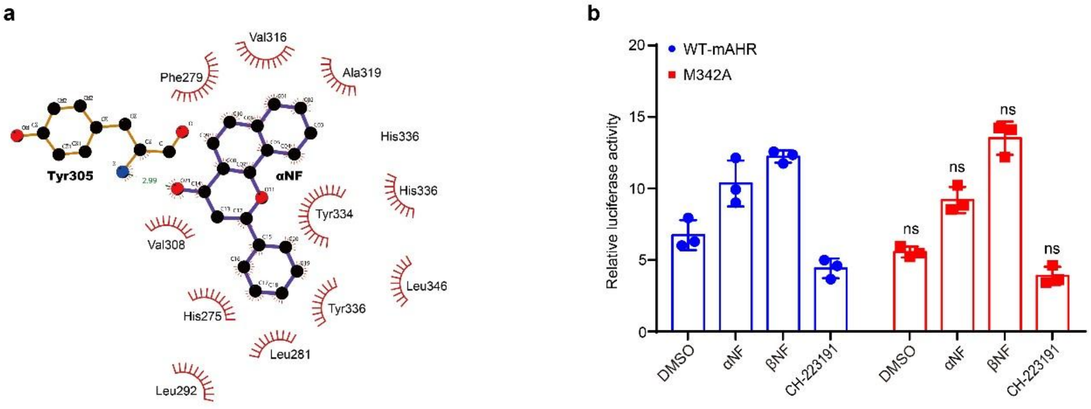


**Supplementary Fig. 3 The binding of αNF to AHR PAS-B. a** LigPlot representation of the interactions between αNF and dAHR PAS-B. **b** DRE luciferase reporter assay examining the effect of the M342A mutation on the transactivation of full-length mAHR in 293T cells. The ligands βNF (agonist), αNF (partial agonist) and CH-223191 (antagonist) were supplied at 500 nM, 1 μM and 1 μM, respectively. n = 3 biological repeats for each group. Data are shown as mean ± SD. *p* values were calculated using a multiple two-sided *t* test with the WT mAHR group treated by the same ligand as a control and presented in the Source Data file. ns: not statistically significant ($p > 0.05$). Source data are provided as a Source Data file.

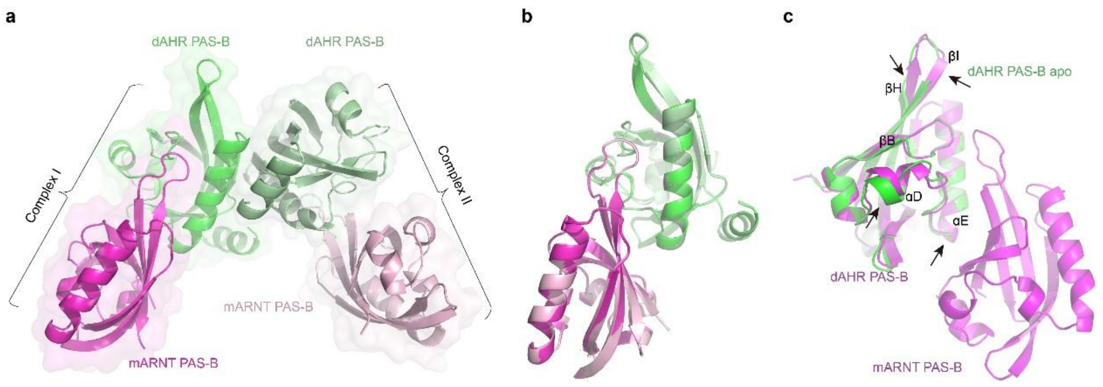


**Supplementary Fig. 4 Crystal structure of dAHR PAS-B:mARNT complex. a** The asymmetric unit of the complex crystal. **b** Superimposition of the two heterodimer complexes (complex I and complex II) in the ASU. **c** Comparison of apo (green) and ARNT-bound (magenta) dAHR PAS-B structures. Conformational differences are indicated by black arrows.

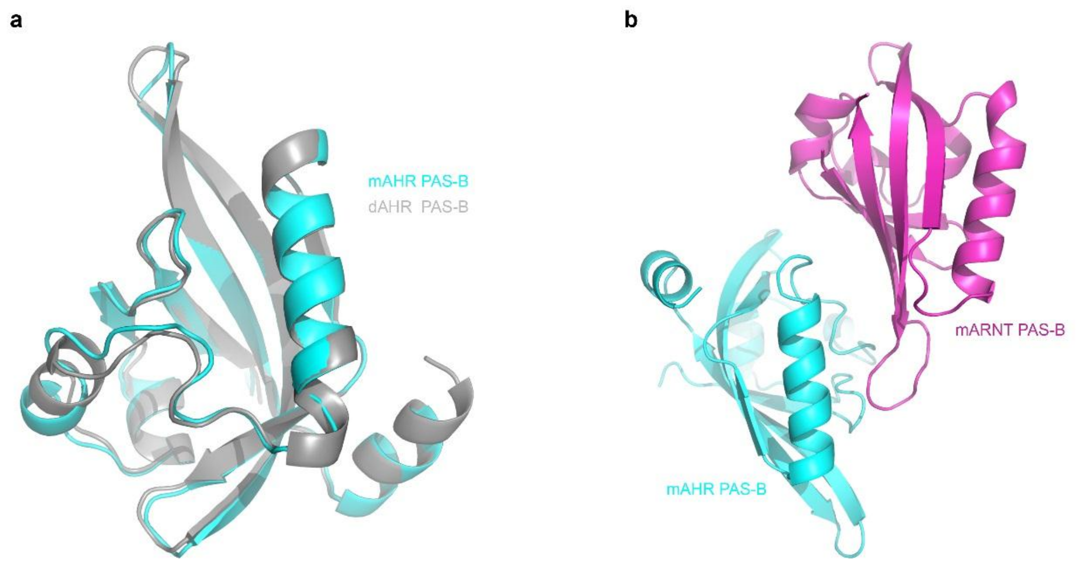


**Supplementary Fig. 5 Structure model mAHR PAS-B and ARNT PAS-B heterodimer. a** mAHR PAS-B model (cyan). The model was generated by the program Modeller with apo dAHR PAS-B structure (gray) used as the single template. **b** mAHR-ARNT PAS-B heterodimer model. The model was generated by roughly overlaying the mAHR PAS-B onto the dAHR PAS-B:mARNT structure.

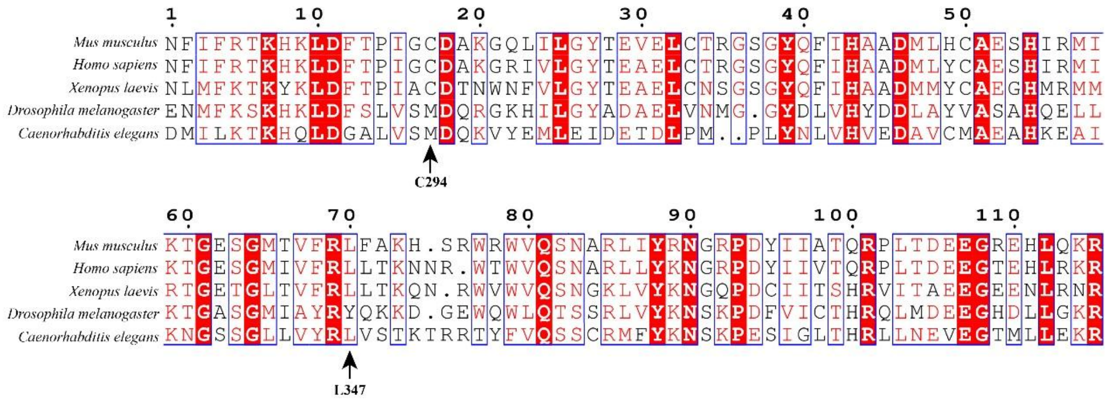

**Supplementary Fig. 6 Multiple sequence alignment of AHR PAS-B domains from different species.**

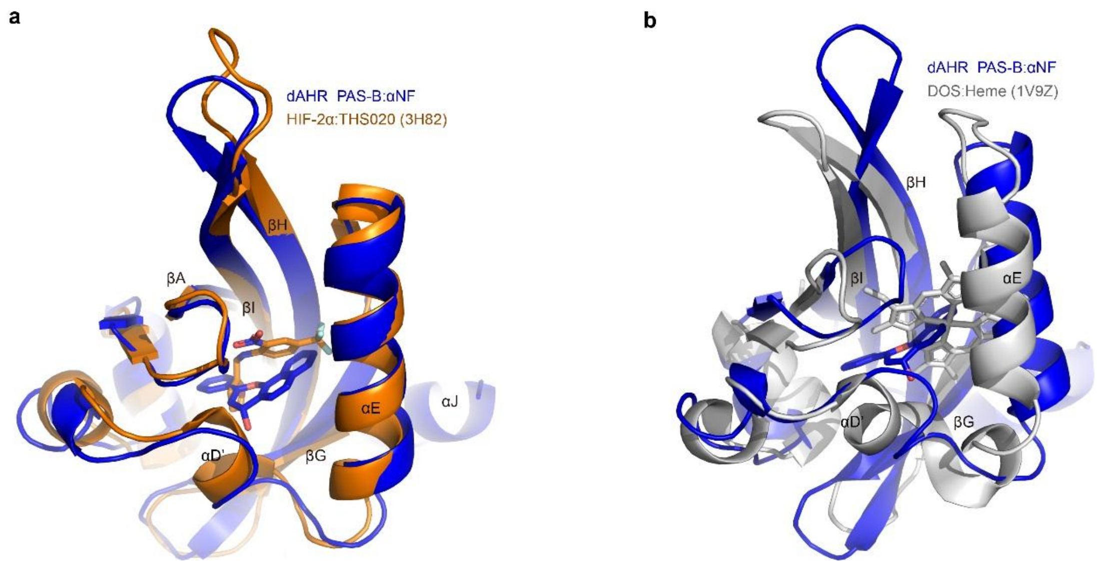


**Supplementary Fig. 7 Ligand binding positions of different PAS proteins. a** Comparison of ligand binding poses of dAHR (blue) and human HIF-2α (orange, PDB code 3H82). **b** Ligand binding poses of dAHR and *Escherichia coli* DOS (grey, PDB code 1V9Z).

## Supplementary Table 1 Data collection and refinement statistics.

| | dAHR PAS-B apo | dAHR PAS-B:αNF | dAHR PAS-B:ARNT |
|---|---|---|---|
| **Data collection** | | | |
| Resolution (Å) | 31.6-2.6 (2.7-2.6) | 30.2-2.4 (2.5-2.4) | 44.16-2.0 (2.1-2.0) |
| Wavelength (Å) | 1.58 | 0.975 | 0.975 |
| Space group | $P2_12_12_1$ | $P4_12_12$ | $C2_1$ |
| a, b, c (Å) | 34.3, 46.8, 81.7 | 60.2, 60.2, 121.0 | 136.0, 55.6, 77.1 |
| α, β, γ (°) | 90, 90, 90 | 90, 90, 90 | 90, 103.1, 90 |
| Unique reflections | 4172 (255) | 9195 (851) | 36452 (3021) |
| Redundancy | 10.7 (2.2) | 23.2 (16.1) | 6.8 (6.1) |
| Completeness (%) | 95.1 (55) | 99.9 (99.8) | 96.8 (96.3) |
| Mean I/sigma (I) | 17.7 (1.4) | 16.3 (2.4) | 16.5 (2.6) |
| **Refinement** | | | |
| R-work / R-free | 0.21 (0.23) /0.26 (0.29) | 0.23 (0.31) /0.27 (0.36) | 0.18 (0.23) / 0.22 (0.28) |
| NO. of non-hydrogen atoms | 915 | 1896 | 3990 |
| Macromolecules | 904 | 1806 | 3554 |
| Ligands | 0 | 42 | 0 |
| Solvent | 11 | 48 | 436 |
| Protein residues | 114 | 226 | 432 |
| RMS (bonds) | 0.007 | 0.004 | 0.006 |
| RMS (angles) | 0.51 | 0.70 | 0.83 |
| Clash score | 6.8 | 5.6 | 10.1 |
| Ramachandran favored (%) | 98 | 97 | 97 |
| Ramachandran outliers (%) | 1.8 | 0 | 2.8 |
| Rotamer outliers (%) | 2.2 | 1.1 | 2.8 |
| Average B-factor | 38.5 | 44.1 | 31.8 |
