# Supplementary material for: Structural insight into the ligand binding mechanism of aryl hydrocarbon receptor
Source: Nat Commun. 2022 Oct 20;13:6234. doi: 10.1038/s41467-022-33858-w (PMC9585082; doi:10.1038/s41467-022-33858-w)
Supplement: Supplementary file 2 — Reporting Summary [file 41467_2022_33858_MOESM2_ESM.pdf]

nature portfolio

Corresponding author(s): Yongheng Chen

Last updated by author(s): Sep 23, 2022

Reporting Summary

Nature Portfolio wishes to improve the reproducibility of the work that we publish. This form provides structure for consistency and transparency in reporting. For further information on Nature Portfolio policies, see our Editorial Policies and the Editorial Policy Checklist.

Statistics

For all statistical analyses, confirm that the following items are present in the figure legend, table legend, main text, or Methods section.

n/a	Confirmed	
☐	☒	The exact sample size (*n*) for each experimental group/condition, given as a discrete number and unit of measurement
☐	☒	A statement on whether measurements were taken from distinct samples or whether the same sample was measured repeatedly
☐	☒	The statistical test(s) used AND whether they are one- or two-sided<br>*Only common tests should be described solely by name; describe more complex techniques in the Methods section.*
☒	☐	A description of all covariates tested
☒	☐	A description of any assumptions or corrections, such as tests of normality and adjustment for multiple comparisons
☐	☒	A full description of the statistical parameters including central tendency (e.g. means) or other basic estimates (e.g. regression coefficient) AND variation (e.g. standard deviation) or associated estimates of uncertainty (e.g. confidence intervals)
☐	☒	For null hypothesis testing, the test statistic (e.g. *F*, *t*, *r*) with confidence intervals, effect sizes, degrees of freedom and *P* value noted<br>*Give P values as exact values whenever suitable.*
☒	☐	For Bayesian analysis, information on the choice of priors and Markov chain Monte Carlo settings
☒	☐	For hierarchical and complex designs, identification of the appropriate level for tests and full reporting of outcomes
☒	☐	Estimates of effect sizes (e.g. Cohen's *d*, Pearson's *r*), indicating how they were calculated

Our web collection on statistics for biologists contains articles on many of the points above.

Software and code

Policy information about availability of computer code

Data collection	Clustal Omega (version 1.2.2, multiple sequence alignment), MO.Control (version 2.4.2, MST data collection), EnVision Manager (version 1.13.3009.14.09, luciferase reporter data collection).
Data analysis	HKL2000 (version 716.1, x-ray data processing), PHENIX (version 1.10.1-2155, structure determination and refinement), WinCoot (version 0.8.9, atomic model building), GraphPad Prism 7.0 (ELESA and luciferase reporter gene assay data analysis), PyMOL (version 2.3.3, molecular graphics and analyses), Autodock (version 4.2, molecular docking), AutoDockTools (version 1.5.6, molecular docking), Origin8 (size exclusion data process), MO.Affinity Analysis (version 3.0.5, MST binding data analyses), Modeller (version 10.3, single template structure modeling), ESPript (version 3.0, alignment result presentation).

For manuscripts utilizing custom algorithms or software that are central to the research but not yet described in published literature, software must be made available to editors and reviewers. We strongly encourage code deposition in a community repository (e.g. GitHub). See the Nature Portfolio guidelines for submitting code & software for further information.

Data

Policy information about availability of data

All manuscripts must include a data availability statement. This statement should provide the following information, where applicable:

- Accession codes, unique identifiers, or web links for publicly available datasets
- A description of any restrictions on data availability
- For clinical datasets or third party data, please ensure that the statement adheres to our policy

Structure coordinates and map files are deposited into the RSCB Protein Data Bank (PDB) with the following accession codes: PDB ID 7VNA [http://doi.org/10.2210/pdb7VNA/pdb] (apo dAHR PAS-B), PDB ID 7VNH [http://doi.org/10.2210/pdb7VNA/pdb] (aNF-bound dAHR PAS-B), and PDB ID 7VNI [http://doi.org/10.2210/pdb7VNI/pdb] (heterodimer). Structure coordinates used in this study are accessible in PDB under the following accession codes: 3F1O [http://doi.org/10.2210/pdb3F1O/pdb], 6E3U [http://doi.org/10.2210/pdb6E3U/pdb], 4ZP4 [http://doi.org/10.2210/pdb4ZP4/pdb], 4F3L [http://doi.org/10.2210/pdb4F3L/pdb], 5SY5 [http://doi.org/10.2210/pdb5SY5/pdb], 3H82 [http://doi.org/10.2210/pdb3H82/pdb], 1V9Z [http://doi.org/10.2210/pdb1V9Z/pdb]. Source data are provided with this paper.

Human research participants

Policy information about studies involving human research participants and Sex and Gender in Research.

Reporting on sex and gender	N/A
Population characteristics	N/A
Recruitment	N/A
Ethics oversight	N/A

Note that full information on the approval of the study protocol must also be provided in the manuscript.

Field-specific reporting

Please select the one below that is the best fit for your research. If you are not sure, read the appropriate sections before making your selection.

☒ Life sciences ☐ Behavioural & social sciences ☐ Ecological, evolutionary & environmental sciences

For a reference copy of the document with all sections, see nature.com/documents/nr-reporting-summary-flat.pdf

Life sciences study design

All studies must disclose on these points even when the disclosure is negative.

Sample size	Sample sizes are stated in the main text figure legend or methods section and were based on standards in the fields of biochemistry and cell biology. For example, ELISA and Luciferase reporter gene assays consisted of 3 replicates as used in published studies (https://doi.org/10.1016/j.bbagrm.2009.06.003, https://doi.org/10.1038/nbt.2672).
Data exclusions	No data were excluded.
Replication	MST binding assays were based on two biological replications as stated in https://doi.org/10.1016/j.molstruc.2014.03.009 and with similar results. SEC and SDS-PAGE analyses were repeated more than three times with almost identical results.
Randomization	Samples were allocated randomly.
Blinding	Blinding was not used in this study. Data were derived from instrument-based measurement and software-based analysis.

Reporting for specific materials, systems and methods

We require information from authors about some types of materials, experimental systems and methods used in many studies. Here, indicate whether each material, system or method listed is relevant to your study. If you are not sure if a list item applies to your research, read the appropriate section before selecting a response.

Materials & experimental systems

n/a	Involved in the study
☐	☒ Antibodies
☐	☒ Eukaryotic cell lines
☒	☐ Palaeontology and archaeology
☒	☐ Animals and other organisms
☒	☐ Clinical data
☒	☐ Dual use research of concern

Methods

n/a	Involved in the study
☒	☐ ChIP-seq
☒	☐ Flow cytometry
☒	☐ MRI-based neuroimaging

Antibodies

Antibodies used	anti-GST rabbit antibody and anti-rabbit secondary detect antibody are provided in the GST 6xHis-tag ELISA kit (Abcam, ab128573).
Validation	According to the manufacturer's website, these antibodies are validated by western blotting.

Eukaryotic cell lines

Policy information about cell lines and Sex and Gender in Research

Cell line source(s)	HEK293T cell line was purchased from American Type Culture Collection.
Authentication	The cell line was not authenticated.
Mycoplasma contamination	The cells were tested negative for mycoplasma.
Commonly misidentified lines (See ICLAC register)	None were used.
