# Supplementary material for: Structural insight into the ligand binding mechanism of aryl hydrocarbon receptor
Source: Nat Commun. 2022 Oct 20;13:6234. doi: 10.1038/s41467-022-33858-w (PMC9585082; doi:10.1038/s41467-022-33858-w)
Supplement: Supplementary file 3 — Peer Review File [file 41467_2022_33858_MOESM3_ESM.pdf]

nature portfolio

Peer Review File

Structural insight into the ligand binding mechanism of aryl hydrocarbon receptor

Open Access This file is licensed under a Creative Commons Attribution 4.0 International License, which permits use, sharing, adaptation, distribution and reproduction in any medium or format, as long as you give appropriate credit to the original author(s) and the source, provide a link to the Creative Commons license, and indicate if changes were made. In the cases where the authors are anonymous, such as is the case for the reports of anonymous peer reviewers, author attribution should be to 'Anonymous Referee' followed by a clear attribution to the source work. The images or other third party material in this file are included in the article’s Creative Commons license, unless indicated otherwise in a credit line to the material. If material is not included in the article’s Creative Commons license and your intended use is not permitted by statutory regulation or exceeds the permitted use, you will need to obtain permission directly from the copyright holder. To view a copy of this license, visit http://creativecommons.org/licenses/by/4.0/.

Reviewer #1 (Remarks to the Author):

The aryl hydrocarbon receptor (AHR) is a ligand-activated basic helix-loop-helix-Per-ARNT-Sim (bHLH-PAS) domain-containing transcription factor with crucial functions in health and disease. Binding of hydrophobic ligands in the cytosol is thought to induce the release of AHR from chaperones, followed by AHR translocation to the nucleus, hetero-dimerization with ARNT and activation of target genes containing a dioxin response element in their promoter region. While structural data of several related transcription factors and the DNA-binding PAS-A domain of AHR have been reported in the past, the ligand-binding PAS-B domain has so far eluded structural analyses. Here, Dai et al. report the crystal structure of the Drosophila PAS-B domain in the ligand-free state, bound to the antagonist alpha-napthoflavone (αNA) and bound to the PAS-B domain of mouse ARNT. The structural study is accompanied by ligand/domain binding experiments, a luciferase reporter assay with structure-based mutants of mouse AHR, and ligand docking experiments.

The article is well written and the structural and functional data appear sound. Furthermore, despite the available structural data on bHLH-PAS domain proteins, there is still a considerable interest in the ligand-bound AHR structure, since small molecules targeting AHR and in particular the PAS-B domain are in the focus of several drug-screening efforts and a ligand-bound structure could guide rationale drug design. One worry related to the current study is that the transcriptional activity of Drosophila AHR, in contrast to the vertebrate counterparts, is not known to be controlled by ligand-binding. Furthermore, as shown by previous work and also in the current study, the PAS-B domain does not even bind to the majority of known AHR ligands. It is therefore not clear why especially αNA binds to the Drosophila PAS-B domain, what are the consequences for transcriptional activation and, after all, whether the Drosophila PAS-B domain structure at all faithfully reflects the ligand-bound structure of a vertebrate AHR. In my view, additional structure-based characterizations of the Drosophila AHR should be performed to establish this protein as a valid model for a ligand-binding AHR (see below for some suggestions). The ligand-free PAS-B domain structure and the PAS-B AHR-ARNT structures comprise solid data, but due to the many previously reported bHLH-PAS structures, they are not as interesting on their own as the ligand-bound PAS-B domain structure.

Major:

1.) The authors should add an omit map and/or a 2Fo-Fc density map of the ligand to unambiguously demonstrate that the ligand is fully bound to the PAS-B domain structure. I would also suggest adding a more schematic drawing of how the ligand is bound (for example, via ligplot).

2.) Does αNA binding to Drosophila AHR alter transcriptional activity of Drosophila AHR in a luciferase reporter assay as described, for example, in PMID 19560568 ? Can a chimeric construct of mouse AHR, in which the PAS-B domain is replaced by that of Drosophila PAS-B, be activated or inhibited by αNA in a luciferase reporter assay?

3.) Can the Drosophila PAS-B domain be converted to a 'proper' ligand-binding domain by mutating the bulky amino acid at the bottom of the presumed ligand-binding groove (in this case, Y336L/M294C, maybe together with some mutations at the rim of the groove, see Fig. 3d)? Again, at least luciferase reporter assays with a mutant chimeric construct as described in point 2 should be performed. In addition, if the constructs can be expressed in a soluble form, direct ligand binding assay would be desirable.

Minor

Fig. 2/3: Some structural comparisons to other ligand-bound PAS domain structures, such as the PAS-B domain of HIF-2α (PMID 19950993) or the PAS domains of FixL, Dos, or MCP (see PMID 21663441) should be added (maybe in the supplement for a more

spacious representation). This would indicate whether the identified ligand-binding site in AHR is at a canonical or non-canonical position.

Fig. 6a: Could these structures be shown in exactly the same orientation of the ARNT (or BMAL) PAS-B domain? This would allow a better comparison.

Methods
The authors report that only 1 out of 10 tested PAS-B domains from different species was soluble. Please add which PAS-B domain constructs were tested without success.

Rounding: I would suggest rounding all determined binding numbers to the first digit of the error, e.g. 1800 +- 500 nM, not 1770+-460 nM.

Layout/typos:
I would suggest increasing the size of some figures (Fig. 2a-e, 4c, 4d and maybe 6).

Line 134. Therefore, the H-bond contributed by dAHR PAS-B Y334 may NOT (?) be expected when αNA binds to mAHR.

Line 216: These elements underwent a certain degree of expansion – what is exactly meant ? Is this really an 'expansion' of the sceondary structure elements or is the presumed expansion maybe caused by a less accurate model building/secondary structure assignment of the apo state due to the lower resolution of the structure ?

Reviewer #2 (Remarks to the Author):

The work done by Dai and all is remarkable and largely merits to be published in nature Communications. This is the first time that a PAS B domain of AhR (the drosophila AhR, DAhR) is crystallized and the work enables to understand how dAhR PAS B domain is structurally organized and interacts with the AhR heterodimeric partner ARNT.
My only reserve concerns the mode of binding of the ligands. I am a bit skeptical about some of the authors' conclusions. do however agree with them that dAhR is constitutively active unlike AhRs from other species (human, rat) due to a more constrained ligand binding pocket.

My reserves are summarized below.

My first remark concerns the luciferase reporter experiments using dAhR, mAhR WT and mutants. The authors have treated the HEK293T cells for 24 hours. This is a too long treatment for ligands like aNF, bNF, FICZ and BAP. The best time treatment for these ligands (and others like indirubin, indigo, ITE, 3MC) is 6-8 hours. These ligands are metabolised by the CYP1A1 and the potency is always lower in 24 hours than in 5-8 hours. If the authors want to work in 24 hours, they can use dioxin or PCBs (126, 81). These chemicals are more stable and equally potent at 6-8 hours and 24 hours.

My second remarks concern the interpretation of the luciferase assays.

Fig 2E.This experiment did not really support the hypothesis that the mutation M342A enhances the binding affinity of antagonists (aNF and PDM2) on mAhR.
Both WT mAhR and M342A AhR has a basal activity which was not (or very slightly) affected by these 2 antagonists.
On the contrary, it seems that the mutation decreases the basal activity of mAhR. The induction factor between DMSO and bNF is almost 3 on M342A mAHR whereas it is 1.2 with WT mAhR. Curiously, the induction factor of bNF on WT mAhR is almost 2 in figure 3b. Are the two experiments done differently ? Could the differences between the two experiments be due to the more or less degradation of bNF in 24 hours.

To be certain, that the affinity for the antagonists will be increase by the M342A mutation, it will be more appropriate to treat the cells with bNF at conditions where this chemical was able to increase the basal activity of AhR (at 200 nM for 5-8 hours) or at a higher concentrations if the treatment is 24 hours) and treat the cells with different concentrations of antagonists. If the mutation increase the affinity for the antagonists, the IC50 of these antagonists will be lower for the mutant mAhR.

I am also disturbed by the fact that aNF bind but does not modulate (even slightly) the constitutive activity of dAhR. I have expected that aNF would decrease (inverse agonist) or increase (agonist) the constitutive basal activity of dAhR. Did the authors test several concentration of bNF (0.1 to 10 microM) ?

Fig 3B.
Again, I am not sure that this experiment fully supports the hypothesis that the double mutation L347Y/C294M strongly decreases the agonistic effect of all the bNF, FICZ and BAP agonists. Notably, FICZ increases by a 2-fold factor the basal activity of both WT and L347Y/C294M mAhR. However, the mutation seems to decrease the basal activity of mAhR by a 2-fold factor.

Fig 5b.
To my opinion, this experiment did not fully support the hypothesis that the mutation Y316A strongly decrease the basal activity of mAhR. On the contrary, it strongly decreases the luciferase activity in presence of AhR agonists.

In conclusion, my reserves concern mainly the interpretation of the luciferase reporter assays and the modulation of the PAS B domain activity by the mutations. Again the other points of the work, the structure of the dAhR PASB domain, the mode of interaction with ARNT are very strong and largely merit to be published in Nature Communications.

Reviewer #3 (Remarks to the Author):

In this paper the Authors present data that constitute important advancements in the understanding of the Aryl Hydrocarbon Receptor (AHR) structure and mechanism. Particularly relevant is the deposition of the first crystal structures of the AHR PAS-B domain, that is involved both in ligand binding and dimerization with ARNT. In past studies, the expression of the AHR PAS-B domains of different species (including human and mouse) resulted only in aggregated and/or insoluble protein precipitates, and this hampered the possibility of obtaining structural insights on this domain as well as of studying the ligand-induced activation mechanism of the AHR. Here the Authors find that the PAS-B domain of Drosophila AHR (dAHR) is soluble and, on this basis, they can obtain the first experimental structures of an AHR PAS-B.
In addition to the apo form, the manuscript describes depositions of an antagonist-bound structure and of the structure of the dimer with the ARNT PAS-B, although with low resolution. With the contribution of biochemical and cell-based assays, this study give several information about both ligand-binding and dimerization of AHR, that can help in future studies of AHR-targeted drugs.

I think that the manuscript deserves publication, with only a few minor revisions, that are listed below:
1) In the Introduction, the Authors should include a brief description of efforts made by the scientific community over the last 20 years to address the issue of the AHR PAS-B structure and ligand-binding modes by using computational modelling. Some suggested references are: Pandini et al., Biochemistry 2007, 46: 696; Bisson et al., J. Med. Chem. 2009, 52:5635; Motto et. al., J Chem Inf Model. 2011, 51:2868. A comprehensive review can be found in: Bonati et al. Curr Opin Toxicol. 2017, 2:42-49.
2) In the Results section, where the sequence similarities are mentioned (e.g. lines 92,

105), also the sequence identities should be reported.
3) In analyzing the structural similarity with other PAS-B heterodimers, NPAS1/NPAS3 proteins are erroneously reported as NASP1/NASP3 (e.g. lines 257, 264, 267). These names have to be corrected.
4) In the description of the mAHR:mARNT PAS-B heterodimer, that was roughly built by the Authors on the basis of the structure of the dAHR dimer, previous works on the mouse AHR:ARNT PAS-B dimer should be cited and discussed. (See: Corrada et al., Mol Biosyst. 2017, 13:981).

Reviewer #4 (Remarks to the Author):

Aryl hydrocarbon receptor (AHR) is a ligand regulated transcription factor with important roles in xenobiotic metabolism, cellular metabolic responses, as well as in the immune system. Given it can be activated by ligands, a better understanding of the activation process is necessary to not only understand receptor dynamics, but for the development of therapeutics to target AHR for the treatment of various diseases (i.e., immunity, cancer). These insights have been hampered by the lack of crystal structures (or any other 3-D structure) defining the ligand-binding domain of AHR (the PAS-B domain). This group has helped resolve this issue by solving the structure of the Drosophila AHR PAS-B domain – both apo, antagonist bound, and bound to its heterodimeric partner, ARNT. They also use functional assays to determine how specific residues affect the overall activity of this receptor. Collectively, the data give insight into how ligand binding affects receptor dynamics and downstream transcriptional responses.

This is an interesting paper with clear translational potential. The structures give critical insight into how AHR acts transcriptionally. Despite these positives, there are a few issues which detract from the manuscript (outlined below). Many of these are easily addressable and will solidify the data/hypothesis generated in this manuscript.

Major points:
1) Figure 2f – given the fact that the M342A lowers basal responses, the interpretation of this figure needs adjustment. The authors state that M342A mutation had no effect on bNF-stimulated luciferase. However, this is not the case if you compare the M342A DMSO control to bNF treatment. If anything, there is a significant effect on transcriptional activity (meaning it did affect agonist binding). The effects on antagonists were minor. Finally, the effects of these ligands on WT receptor activity were minimal, indicating little response in general occurred. These assays should be redone to generate better windows of response in WT and then see how the mutant affects transcriptional activity.

2) Similar to what is mentioned above – Figure 3b's interpretation needs adjusting. Again, basal transcriptional responses are reduced with the mutant, so again, you need to compare ligand treatment of mutant to DMSO mutant. In doing this, the fold change between DMSO vs drug treatment in the mutant is similar to the fold change observed with WT vs ligand treatments. This changes the authors conclusions. Importantly, how do the individual mutations affect transcriptional responses (i.e., Perform experiments with single mutants)?

3) When determining the binding constant of mARNT PAS-B and dAHR PAS-B, it would be good to mention how this compares to that of the heterodimer formed between human AHR and mouse ARNT. If they are similar, it could help reinforce how conserved this interaction is and/or the overall importance.

4) Figure 5b – all ligands used are those that don't bind to dAHR. Since aNF was the subject of the first part of the paper, including this as a control is necessary.

Methods:
Overall, methods section is well-written and has sufficient detail. However, there are a small number of points that should be clarified.
1) More details are needed for the site-directed mutagenesis (i.e., was a kit used? If so, which one, etc.)

2) For the luciferase assays – authors state that 6h post-transfection, the cells are treated with ligands. Is 6h sufficient to start producing enough protein to form a genuine, specific response?

3) For all luciferase assays – no statistical analysis was performed. This needs to be included.

4) Given point #3 above, there should be a Statistics section with more info needed: types of analysis performed (i.e., ANOVA, students t-test, etc.).

Minor points:
Authors misspelled BMAL1 on line 257

We thank the reviewers for their constructive suggestions, which have helped us to further improve the manuscript. Below are our detailed responses to each of the points raised.

Reviewer #1 (Remarks to the Author):

The aryl hydrocarbon receptor (AHR) is a ligand-activated basic helix-loop-helix-Per-ARNT-Sim (bHLH-PAS) domain-containing transcription factor with crucial functions in health and disease. Binding of hydrophobic ligands in the cytosol is thought to induce the release of AHR from chaperones, followed by AHR translocation to the nucleus, hetero-dimerization with ARNT and activation of target genes containing a dioxin response element in their promoter region. While structural data of several related transcription factors and the DNA-binding PAS-A domain of AHR have been reported in the past, the ligand-binding PAS-B domain has so far eluded structural analyses. Here, Dai et al. report the crystal structure of the Drosophila PAS-B domain in the ligand-free state, bound to the antagonist alpha-napthoflavone (αNA) and bound to the PAS-B domain of mouse ARNT. The structural study is accompanied by ligand/domain binding experiments, a luciferase reporter assay with structure-based mutants of mouse AHR, and ligand docking experiments.

The article is well written and the structural and functional data appear sound. Furthermore, despite the available structural data on bHLH-PAS domain proteins, there is still a considerable interest in the ligand-bound AHR structure, since small molecules targeting AHR and in particular the PAS-B domain are in the focus of several drug-screening efforts and a ligand-bound structure could guide rationale drug design. One worry related to the current study is that the transcriptional activity of Drosophila AHR, in contrast to the vertebrate counterparts, is not known to be controlled by ligand-binding. Furthermore, as shown by previous work and also in the current study, the PAS-B domain does

not even bind to the majority of known AHR ligands. It is therefore not clear why especially αNA binds to the Drosophila PAS-B domain, what are the consequences for transcriptional activation and, after all, whether the Drosophila PAS-B domain structure at all faithfully reflects the ligand-bound structure of a vertebrate AHR. In my view, additional structure-based characterizations of the Drosophila AHR should be performed to establish this protein as a valid model for a ligand-binding AHR (see below for some suggestions). The ligand-free PAS-B domain structure and the PAS-B AHR-ARNT structures comprise solid data, but due to the many previously reported bHLH-PAS structures, they are not as interesting on their own as the ligand-bound PAS-B domain structure.

Major:

1.) The authors should add an omit map and/or a 2Fo-Fc density map of the ligand to unambiguously demonstrate that the ligand is fully bound to the PAS-B domain structure. I would also suggest adding a more schematic drawing of how the ligand is bound (for example, via ligplot).

Response: We thank the reviewer for the suggestion. We have added a 2Fo-Fc density map of the ligand in the revised Fig. 2d. A schematic drawing of how the ligand is bound by dAHR PAS-B is provided in the new Supplementary Fig. 3a.

2.) Does αNA binding to Drosophila AHR alter transcriptional activity of Drosophila AHR in a luciferase reporter assay as described, for example, in PMID 19560568? Can a chimeric construct of mouse AHR, in which the PAS-B domain is replaced by that of Drosophila PAS-B, be activated or inhibited by αNA in a luciferase reporter assay?

Response: We thank the reviewer for the comment. We have performed luciferase reporter assays with full-length dAHR. dAHR showed little activity in

HEK293T cells, and we could not observe that the presence of αNF affected dAHR activity. This result is similar to the previous report that dAHR has little or no activity in Hepa-1c1c7 cells and could not be induced by TCDD. We also performed luciferase assays for a chimeric mAHR with its PAS-B domain replaced by that of dAHR (mAHR-dPB). Compared to mAHR, mAHR-dPB has a higher basal activity. Different concentrations of αNF (0.1, 0.5, 1 and 10 μM) do not affect its transcriptional activity. Therefore, we think that although αNF can bind dAHR PAS-B, it is not able to alter the transcriptional activity of mAHR-dPB. Based on these results, we infer that αNF may not be able to modulate the activity of constitutively activated dAHR either. These results have been added to the Results section, and the relevant figures are shown in Supplementary Fig. 2.

3.) Can the Drosophila PAS-B domain be converted to a 'proper' ligand-binding domain by mutating the bulky amino acid at the bottom of the presumed ligand-binding groove (in this case, Y336L/M294C, maybe together with some mutations at the rim of the groove, see Fig. 3d)? Again, at least luciferase reporter assays with a mutant chimeric construct as described in point 2 should be performed. In addition, if the constructs can be expressed in a soluble form, direct ligand binding assay would be desirable.

Response: Thank you for your insightful suggestions. We constructed and purified the dAHR PAS-B M284C/Y336L mutant. MST binding assay showed that M284C/Y336L mutant gained the ability to bind βNF and FICZ, with estimated Kd values around 400 and 800 nM, respectively (Fig. 3b). On the other hand, we also introduced the M284C/Y336L mutation into mAHR-dPB (mAHR-dPB-CL) and performed luciferase assays. Unexpectedly, this mutation dramatically decreased mAHR-dPB transcription activity. The presence of βNF or FICZ only slightly increased its activity, possibly due to the activation of endogenous hAHR in HEK293T cells (please refer to the below

picture for detail).

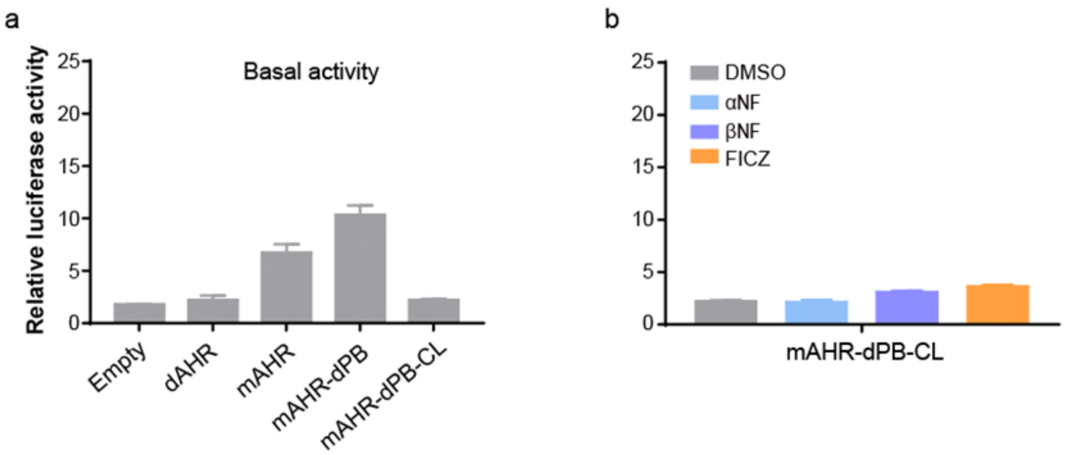

Minor

Fig. 2/3: Some structural comparisons to other ligand-bound PAS domain structures, such as the PAS-B domain of HIF-2α (PMID 19950993) or the PAS domains of FixL, Dos, or MCP (see PMID 21663441) should be added (maybe in the supplement for a more spacious representation). This would indicate whether the identified ligand-binding site in AHR is at a canonical or non-canonical position.

Response: As per the reviewer's suggestion, we compared our structure with the ligand-bound HIF-2α (PDB code, 3H82) and Dos (PDB code, 1V9Z) structures. αNF binds to the dAHR PAS-B domain at a pocket near αE, βH, βG and αD' The binding of the artificial ligand THS020 to HIF-2α and Heme to E. coli Dos also occurred at a similar location. Thus, the binding of αNF in dAHR PAS-B is at a canonical position in the pocket of the PAS domain. We have discussed this point in the Discussion section, and the relevant figures are presented in Supplementary Fig. 7.

Fig. 6a: Could these structures be shown in exactly the same orientation of the ARNT (or BMAL) PAS-B domain? This would allow a better comparison.

Response: Thank you for the suggestion. We have revised Fig. 6a to ensure that all structures placed with ARNT or BMAL PAS-B are presented in the same orientation.

Methods

The authors report that only 1 out of 10 tested PAS-B domains from different species was soluble. Please add which PAS-B domain constructs were tested without success.

Response: As per the reviewer's comment, we have provided the information of all 12 tested PAS-B domains in the Methods section in the revised manuscript.

Rounding: I would suggest rounding all determined binding numbers to the first digit of the error, e.g. 1800 +- 500 nM, not 1770+-460 nM.

Response: Thank you for the comment. These values are changed accordingly.

Layout/typos:

I would suggest increasing the size of some figures (Fig. 2a-e, 4c, 4d and maybe 6).

Response: As per the reviewer's suggestion, we have increased the size of these figures (including Fig. 2a-e, 4c, 4d and Fig. 6).

Line 134. Therefore, the H-bond contributed by dAHR PAS-B Y334 may NOT (?) be expected when αNA binds to mAHR.

Response: We thank the reviewer for carefully reading. We have corrected this sentence.

Line 216: These elements underwent a certain degree of expansion – what is exactly meant? Is this really an 'expansion' of the secondary structure elements or is the presumed expansion maybe caused by a less accurate model building/secondary structure assignment of the apo state due to the lower resolution of the structure?

Response: We thank the reviewer for pointing out this concern. We fully agree with the reviewer that these observed differences could be caused by resolution limits or different crystal packing environments. We have rewritten this paragraph in the revised manuscript.

Reviewer #2 (Remarks to the Author):

The work done by Dai and all is remarkable and largely merits to be published in nature Communications. This is the first time that a PAS B domain of AhR (the drosophila AhR, DAhR) is crystallized and the work enables to understand how dAhR PAS B domain is structurally organized and interacts with the AhR heterodimeric partner ARNT.

My only reserve concerns the mode of binding of the ligands. I am a bit skeptical about some of the authors' conclusions. do however agree with them that dAhR is constitutively active unlike AhRs from other species (human, rat) due to a more constrained ligand binding pocket.

My reserves are summarized below.

My first remark concerns the luciferase reporter experiments using dAhR, mAhR WT and mutants. The authors have treated the HEK293T cells for 24 hours. This is a too long treatment for ligands like aNF, bNF, FICZ and BAP. The best time treatment for these ligands (and others like indirubin, indigo, ITE, 3MC) is 6-8 hours. These ligands are metabolized by the CYP1A1 and the potency is always lower in 24 hours than in 5-8 hours. If the authors want to

work in 24 hours, they can use dioxin or PCBs (126, 81). These chemicals are more stable and equally potent at 6-8 hours and 24 hours.

Response: We thank the reviewer for pointing out this concern. We agree with the reviewer that 24 hours may be too long for these ligands we used. We screened the posttransfection time (6, 12, 20, 24 hours) and ligand treatment time (4, 6, 10 and 24 hours) to generate better response windows. The resulting response levels for most tested ligands were maximum (2-3-fold enrichment compared to DMSO treatment for tested agonists) at the combination of 20 hours posttransfection and 10 hours of ligand treatment. We redid all luciferase reporter gene assays (Fig. 5b, s2 and s3a) under this condition in the revised manuscript.

My second remarks concern the interpretation of the luciferase assays.

Fig 2E.This experiment did not really support the hypothesis that the mutation M342A enhances the binding affinity of antagonists (aNF and PDM2) on mAhR. Both WT mAhR and M342A AhR has a basal activity which was not (or very slightly) affected by these 2 antagonists. On the contrary, it seems that the mutation decreases the basal activity of mAhR. The induction factor between DMSO and bNF is almost 3 on M342A mAHR whereas it is 1.2 with WT mAhR. Curiously, the induction factor of bNF on WT mAhR is almost 2 in figure 3b. Are the two experiments done differently? Could the differences between the two experiments be due to the more or less degradation of bNF in 24 hours.

Response: Thank you for pointing out these concerns. We carefully redid the luciferase assay for mAHR M342A mutant following the refined protocol. This time, we could not observe significant luciferase activity change for M342A in the absence or presence of different ligands (αNF, βNF and CH223191) compared to the WT mAHR. The new results are presented in Supplementary Fig. 3b. Therefore, we have changed our conclusion in the revised manuscript.

To be certain, that the affinity for the antagonists will be increase by the M342A mutation, it will be more appropriate to treat the cells with bNF at conditions where this chemical was able to increase the basal activity of AhR (at 200 nM for 5-8 hours) or at a higher concentrations if the treatment is 24 hours) and treat the cells with different concentrations of antagonists. If the mutation increase the affinity for the antagonists, the IC50 of these antagonists will be lower for the mutant mAhR.

Response: Thank you for the suggestion. We have redone the luciferase assay for M342A and found no significant difference in luciferase activity compared to the WT mAHR. We have adjusted our conclusion in the revised manuscript.

I am also disturbed by the fact that aNF bind but does not modulate (even slightly) the constitutive activity of dAhR. I have expected that aNF would decrease (inverse agonist) or increase (agonist) the constitutive basal activity of dAhR. Did the authors test several concentration of bNF (0.1 to 10 microM) ?

Response: We thank the reviewer for this valuable comment and suggestion. We performed luciferase reporter assays with full-length dAHR. dAHR showed little activity in HEK293T cells, and we could not observe that the presence of αNF affected dAHR activity. This result is similar to the previous report that dAHR has little or no activity in Hepa-1c1c7 cells and could not be induced by TCDD. We also performed luciferase assays for a chimeric mAHR with its PAS-B domain replaced by that of dAHR (mAHR-dPB). Compared to mAHR, mAHR-dPB has a higher basal activity. Different concentrations of αNF (0.1, 0.5, 1 and 10 μM) do not affect its transcriptional activity. Therefore, we think that although αNF can bind dAHR PAS-B, it is not able to alter the transcriptional activity of mAHR-dPB. Based on these results, we infer that αNF may not be able to modulate the activity of constitutively activated dAHR

either. These results have been added to the Results section, and the relevant figures are shown in Supplementary Fig. 2.

Fig 3B. Again, I am not sure that this experiment fully supports the hypothesis that the double mutation L347Y/C294M strongly decreases the agonistic effect of all the βNF, FICZ and BAP agonists. Notably, FICZ increases by a 2-fold factor the basal activity of both WT and L347Y/C294M mAhR. However, the mutation seems to decrease the basal activity of mAhR by a 2-fold factor.

Response: Thank you for pointing out this concern. We have redone this experiment. In addition, by following the suggestion of reviewer #3, we also constructed L347Y and C294M single-site mutants. Compared to the WT protein, the ligand induction and the basal activity of L347Y and C294 M/L347Y were largely decreased. When comparing the induction folds (ligand treatment vs DMSO treatment), L347 and C294M/L347 showed unexpectedly slightly increased induction folds (please refer to the picture below for detail). We think the decrease in basal activity may be due to the diminished activation by endogenous ligands. To avoid over-interpreting this data, we removed it in the revised manuscript.

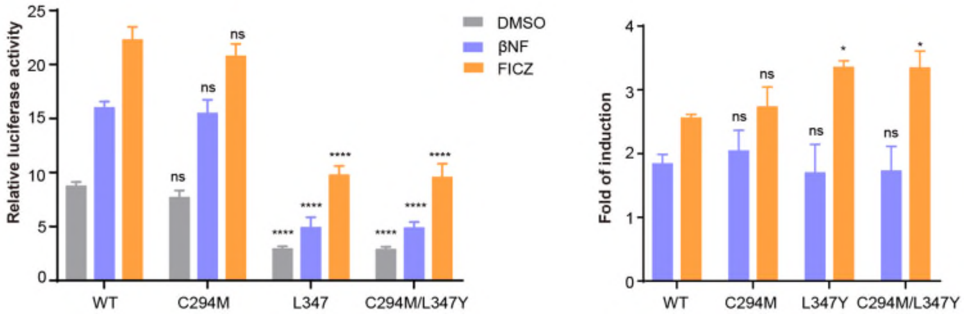

On the other hand, we purified the dAHR PAS-B variant bearing the M284C/Y336L double-site mutation and evaluated its ligand binding ability by MST. M284C/Y336L gained the ability to bind βNF and FICZ with Kd values of approximately 400 nM and 800 nM, respectively (Fig. 3b). Therefore, we think that these two residues of dAHR play an important role in blocking the binding

of AHR ligands.

Fig 5b. To my opinion, this experiment did not fully support the hypothesis that the mutation Y316A strongly decrease the basal activity of mAhR. On the contrary, it strongly decreases the luciferase activity in presence of AhR agonists.

Response: Thank you for the comment. We have redone this experiment. Both the basal activity and induction activity of Y316A were strongly decreased. We then compared the fold increase in luciferase activity due to ligand treatment (ligand treatment divided by DMSO treatment). Y316A showed a reduced, while A321Y showed an increased induction fold.

In conclusion, my reserves concern mainly the interpretation of the luciferase reporter assays and the modulation of the PAS B domain activity by the mutations. Again the other points of the work, the structure of the dAhR PASB domain, the mode of interaction with ARNT are very strong and largely merit to be published in Nature Communications.

We thank the reviewer again for his or her valuable comments and suggestions.

Reviewer #3 (Remarks to the Author):

In this paper the Authors present data that constitute important advancements in the understanding of the Aryl Hydrocarbon Receptor (AHR) structure and mechanism. Particularly relevant is the deposition of the first crystal structures of the AHR PAS-B domain, that is involved both in ligand binding and dimerization with ARNT. In past studies, the expression of the AHR PAS-B domains of different species (including human and mouse) resulted only in aggregated and/or insoluble protein precipitates, and this hampered the possibility of obtaining structural insights on this domain as well as of studying

the ligand-induced activation mechanism of the AHR. Here the Authors find that the PAS-B domain of Drosophila AHR (dAHR) is soluble and, on this basis, they can obtain the first experimental structures of an AHR PAS-B.

In addition to the apo form, the manuscript describes depositions of an antagonist-bound structure and of the structure of the dimer with the ARNT PAS-B, although with low resolution. With the contribution of biochemical and cell-based assays, this study give several information about both ligand-binding and dimerization of AHR, that can help in future studies of AHR-targeted drugs.

I think that the manuscript deserves publication, with only a few minor revisions, that are listed below:

1) In the Introduction, the Authors should include a brief description of efforts made by the scientific community over the last 20 years to address the issue of the AHR PAS-B structure and ligand-binding modes by using computational modelling. Some suggested references are: Pandini et al., Biochemistry 2007, 46: 696; Bisson et al., J. Med. Chem. 2009, 52:5635; Motto et. al., J Chem Inf Model. 2011, 51:2868. A comprehensive review can be found in: Bonati et al. Curr Opin Toxicol. 2017, 2:42-49.

Response: Thank you for the comment. In the revised manuscript, this point has been added, and relevant references have been cited.

2) In the Results section, where the sequence similarities are mentioned (e.g. lines 92, 105), also the sequence identities should be reported.

Response: Thank you for the suggestion. The sequence identities have been provided in the text and corresponding figure (Fig. 1a) in the revised manuscript.

3) In analyzing the structural similarity with other PAS-B heterodimers, NPAS1/NPAS3 proteins are erroneously reported as NASP1/NASP3 (e.g. lines 257, 264, 267). These names have to be corrected.

Response: Thank you for pointing out these mistakes. We have fixed them in the revised manuscript.

4) In the description of the mAHR:mARNT PAS-B heterodimer, that was roughly built by the Authors on the basis of the structure of the dAHR dimer, previous works on the mouse AHR:ARNT PAS-B dimer should be cited and discussed. (See: Corrada et al., Mol Biosyst. 2017, 13:981).

Response: Thank you for the comment. We have added this point, and relevant references were cited in the revised manuscript.

Reviewer #4 (Remarks to the Author):

Aryl hydrocarbon receptor (AHR) is a ligand regulated transcription factor with important roles in xenobiotic metabolism, cellular metabolic responses, as well as in the immune system. Given it can be activated by ligands, a better understanding of the activation process is necessary to not only understand receptor dynamics, but for the development of therapeutics to target AHR for the treatment of various diseases (i.e., immunity, cancer). These insights have been hampered by the lack of crystal structures (or any other 3-D structure) defining the ligand-binding domain of AHR (the PAS-B domain). This group has helped resolve this issue by solving the structure of the Drosophila AHR PAS-B domain – both apo, antagonist bound, and bound to its heterodimeric partner, ARNT. They also use functional assays to determine how specific residues affect the overall activity of this receptor. Collectively, the data give insight into how ligand binding affects receptor dynamics and downstream transcriptional responses.

This is an interesting paper with clear translational potential. The structures give critical insight into how AHR acts transcriptionally. Despite these positives, there are a few issues which detract from the manuscript (outlined below). Many of these are easily addressable and will solidify the data/hypothesis generated in this manuscript.

Major points:

1) Figure 2f – given the fact that the M342A lowers basal responses, the interpretation of this figure needs adjustment. The authors state that M342A mutation had no effect on bNF-stimulated luciferase. However, this is not the case if you compare the M342A DMSO control to bNF treatment. If anything, there is a significant effect on transcriptional activity (meaning it did affect agonist binding). The effects on antagonists were minor. Finally, the effects of these ligands on WT receptor activity were minimal, indicating little response in general occurred. These assays should be redone to generate better windows of response in WT and then see how the mutant affects transcriptional activity.

Response: We thank the review very much for the insightful comment. We screened the posttransfection time (6, 12, 20, 24 hours) and ligand treatment time (4, 6, 10 and 24 hours) to generate better response windows. The response for most tested ligands reached a maximum level (2-3-fold) at the combination of 20 hours posttransfection and 10 hours of ligand treatment. We then redid all our luciferase reporter gene assays under this condition. According to the new result, no significant luciferase difference was observed between WT and M342A. We have adjusted our conclusion in the revised manuscript.

2) Similar to what is mentioned above – Figure 3b's interpretation needs adjusting. Again, basal transcriptional responses are reduced with the mutant, so again, you need to compare ligand treatment of mutant to DMSO mutant. In

doing this, the fold change between DMSO vs drug treatment in the mutant is similar to the fold change observed with WT vs ligand treatments. This changes the authors conclusions. Importantly, how do the individual mutations affect transcriptional responses (i.e., Perform experiments with single mutants)?

Response: Thank you for the comment and suggestion. We have constructed mAHR L347 and C294M single-site mutants and repeated the luciferase reporter assays. Compared to the WT protein, the ligand induction as well as the basal activity of L347Y and C294 M/L347Y were largely decreased. When comparing the induction folds (ligand treatment vs DMSO treatment), L347 and C294M/L347 showed unexpectedly slightly increased induction folds (please refer to the picture below for detail). We think the decrease in basal activity may be due to the diminished activation by endogenous ligands. To avoid over-interpreting this data, we decided to remove it in the revised manuscript.

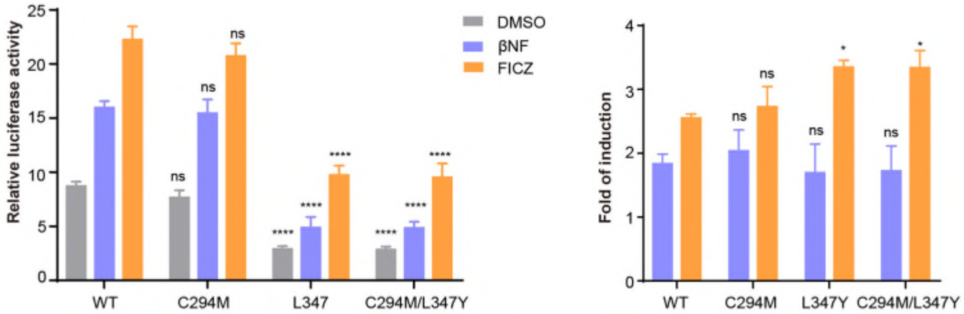

3) When determining the binding constant of mARNT PAS-B and dAHR PAS-B, it would be good to mention how this compares to that of the heterodimer formed between human AHR and mouse ARNT. If they are similar, it could help reinforce how conserved this interaction is and/or the overall importance.

Response: Thank you for the suggestion. We analyzed dAHR PAS-B residues participating in the interaction with mARNT PAS-B, 9 out of 11 are conserved in mAHR and hAHR (Fig. 4e). These observations suggest that the AHR-ARNT

PAS-B interaction pattern may be highly conserved among these species. We have mentioned this point information in the revised manuscript.

4) Figure 5b – all ligands used are those that don't bind to dAHR. Since aNF was the subject of the first part of the paper, including this as a control is necessary.

Response: Thank you for the comment. We redid this assay with αNF included.

Methods:

Overall, methods section is well-written and has sufficient detail. However, there are a small number of points that should be clarified.

1) More details are needed for the site-directed mutagenesis (i.e., was a kit used? If so, which one, etc.)

Response: Thank you for the comment. All site-directed mutants used in this manuscript were generated with the KOD-Plus-Mutagenesis kit (TOYOBO, SMK-101). We have detailed this aspect in the Methods section of the revised manuscript.

2) For the luciferase assays – authors state that 6h post-transfection, the cells are treated with ligands. Is 6h sufficient to start producing enough protein to form a genuine, specific response?

Response: Thank you for pointing out this concern. We screened the posttransfection time (6, 12, 20, 24 hours) and ligand treatment time (4, 6, 10 and 24 hours) to generate better response windows. The response for most tested ligands reached a maximum level (2-3-fold) at the combination of 20 hours posttransfection and 10 hours of ligand treatment. We then redid all our luciferase reporter gene assays under this condition.

3) For all luciferase assays – no statistical analysis was performed. This needs to be included.

Response: Thank you for the comment. We have performed statistical analysis for all luciferase assays in the revised manuscript.

4) Given point #3 above, there should be a Statistics section with more info needed: types of analysis performed (i.e., ANOVA, students t-test, etc.).

Response: Thank you for the comment. Statistics are provided in the revised manuscript.

Minor points:

Authors misspelled BMAL1 on line 257

Response: We have corrected the error.

Reviewer #1 (Remarks to the Author):

My concerns have been satisfactorily addressed. In my view, this is an important and well conducted study that merits publication in Nature Communication. Some more suggestions, mostly for minor text changes and typos below.

L72: has not been solved, since the human and mouse PAS-B domains could not be expressed in a soluble form and/or were aggregating.

L74: characteristics

L93: 63%, 47% and 44% amino acid identity.

L126 in a ligand-independent manner

Line 127: MST bindings assays demonstrated that … in vitro.
This sentence is redundant with the paragraph before and could be removed.

L 148: electron density

L160-166: Although maybe not essential, an in-vitro ligand-binding assay for M342A would be neat to complete the dataset (if added, there is no need for me to see this experiment).

Line 259 showed increased induction levels (Fig. 5b, lower panels). These results are consistent with … (e.g. remove 'higly')

Line 295: According, electrostatic interactions and the number of interface residues …

Line 748: Polar interactions (instead of 'electronic interactions')

We thank the reviewer for his or her careful reading and valuable suggestions. All concerns raised by the reviewer have been addressed.

Reviewer #1 (Remarks to the Author):

My concerns have been satisfactorily addressed. In my view, this is an important and well conducted study that merits publication in Nature Communication. Some more suggestions, mostly for minor text changes and typos below.

1) L72: has not been solved, since the human and mouse PAS-B domains could not be expressed in a soluble form and/or were aggregating.

Response: Thanks for the suggestion. We have revised this sentence.

2) L74: characteristics

Response: We thank the reviewer for careful reading. We have corrected this typo.

3) L93: 63%, 47% and 44% amino acid identity.

Response: This sentence has been revised accordingly.

4) L126 in a ligand-independent manner

Response: This sentence has been revised.

5) Line 127: MST bindings assays demonstrated that … in vitro. This sentence is redundant with the paragraph before and could be removed.

Response: Thanks for the comments. Changes have been made accordingly.

6) L 148: electron density

Response: Thanks for pointing out this mistake for us. We have corrected it.

7) L160-166: Although maybe not essential, an in-vitro ligand-binding assay for M342A would be neat to complete the dataset (if added, there is no need for me to see this experiment).

Response: Thanks for the suggestion. Since mAHR dPAS-B cannot be solubly expressed in *E. coli*, we cannot do in vitro ligand-binding assays for the M342A mutant.

8) Line 259 showed increased induction levels (Fig. 5b, lower panels). These results are consistent with … (e.g. remove 'higly')

Response: We thank the reviewer for his or her valuable suggestions. Changes have been made accordingly.

9) Line 295: According, electrostatic interactions and the number of interface residues …

Response: Thanks for the suggestion. We have revised this sentence accordingly.

10) Line 748: Polar interactions (instead of 'electronic interactions')

Response: We have changed 'Electronic interactions' to 'Polar interactions' as suggested by the reviewer.
